# Noise filtering tradeoffs in spatial gradient sensing and cell polarization response

**DOI:** 10.1186/1752-0509-5-196

**Published:** 2011-12-13

**Authors:** Ching-Shan Chou, Lee Bardwell, Qing Nie, Tau-Mu Yi

**Affiliations:** 1 Department of Mathematics, The Ohio State University, Columbus, OH 43210, USA; 2 Center for Mathematical and Computational Biology Center for Complex Biological Systems Department of Mathematics University of California, Irvine Irvine, CA 92697, USA; 3 Center for Complex Biological Systems Department of Developmental and Cell Biology University of California, Irvine Irvine, CA 92697, USA; 4 Tau-Mu Yi Assistant Professor of Developmental and Cell Biology 2011 Biological Sciences III University of California, Irvine Irvine, CA 92697, USA

**Keywords:** Noise/gradient-sensing/G-protein/cell, polarity/yeast mating

## Abstract

**Background:**

Cells sense chemical spatial gradients and respond by polarizing internal components. This process can be disrupted by gradient noise caused by fluctuations in chemical concentration.

**Results:**

We investigated how external gradient noise affects spatial sensing and response focusing on noise-filtering and the resultant tradeoffs. First, using a coarse-grained mathematical model of gradient-sensing and cell polarity, we characterized three negative consequences of noise: Inhibition of the extent of polarization, degradation of directional accuracy, and production of a noisy output polarization. Next, we explored filtering strategies and discovered that a combination of positive feedback, multiple signaling stages, and time-averaging produced good results. There was an important tradeoff, however, because filtering resulted in slower polarization. Simulations demonstrated that a two-stage filter-amplifier resulted in a balanced outcome. Then, we analyzed the effect of noise on a mechanistic model of yeast cell polarization in response to gradients of mating pheromone. This analysis showed that yeast cells likely also combine the above three filtering mechanisms into a filter-amplifier structure to achieve impressive spatial-noise tolerance, but with the consequence of a slow response time. Further investigation of the amplifier architecture revealed two positive feedback loops, a fast inner and a slow outer, both of which contributed to noise-tolerant polarization. This model also made specific predictions about how orientation performance depended upon the ratio between the gradient slope (signal) and the noise variance. To test these predictions, we performed microfluidics experiments measuring the ability of yeast cells to orient to shallow gradients of mating pheromone. The results of these experiments agreed well with the modeling predictions, demonstrating that yeast cells can sense gradients shallower than 0.1% μm^-1^, approximately a single receptor-ligand molecule difference between front and back, on par with motile eukaryotic cells.

**Conclusions:**

Spatial noise impedes the extent, accuracy, and smoothness of cell polarization. A combined filtering strategy implemented by a filter-amplifier architecture with slow dynamics was effective. Modeling and experimental data suggest that yeast cells employ these elaborate mechanisms to filter gradient noise resulting in a slow but relatively accurate polarization response.

## Background

Cells sense and respond to external cues in a noisy environment [1]. These stimuli include light, nutrients, repellents, etc. Cells must filter the signal from noise, process the relevant information, and then mount the appropriate response (e.g. moving, making a projection). For chemical signals such as an attractant, a cell measures not only the absolute concentration but also the changes in concentration with respect to time or space [2,3]. Noise fluctuations impede the accurate assessment of these signal changes [4].

In bacterial chemotaxis, motile bacteria cells choose the appropriate direction to move by sampling the concentration of attractant at different time points, calculating the temporal difference, and deciding to run in a straight path or to change direction. Berg and Purcell [5] identified diffusive noise (i.e. the fluctuating numbers of ligand molecules diffusing into the vicinity of the cell) as a critical challenge for this system. Several authors [5-7] have determined the properties of an optimal filter for separating signal from noise in temporal sensing.

**Figure 1:**
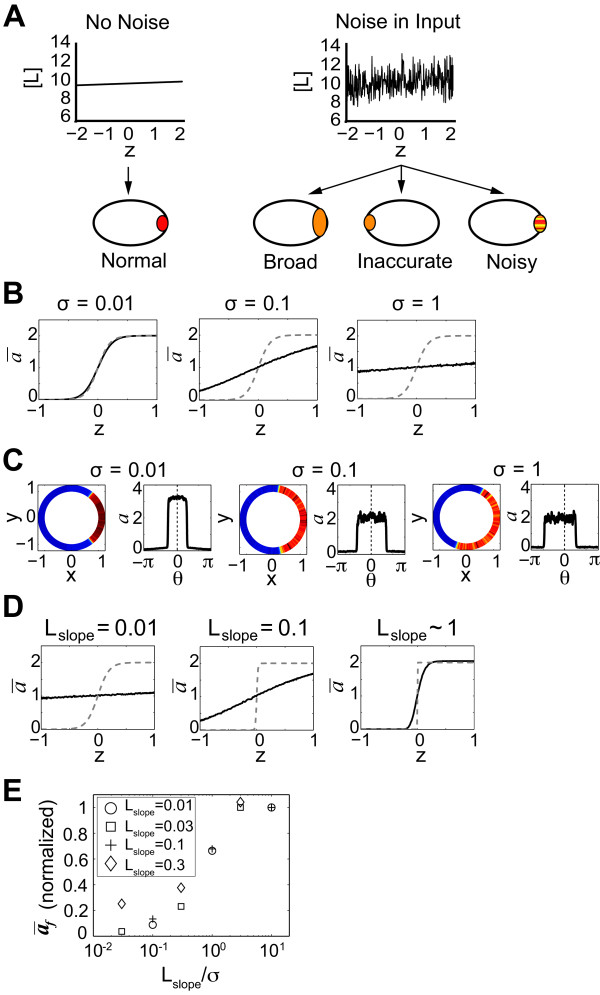
**Effects of spatial noise on cell polarity**. **(A) **Diagram showing input chemical gradient [L] without noise (left) and with noise (right) plotted against axial length *z*. Polarity response is represented by localization of the red species. Spatial input noise makes sensing and responding to the gradient more difficult affecting the extent, directional accuracy, and smoothness of the output polarization. **(B) **Spatial noise inhibits cell polarity. Increasing the magnitude of the noise (*σ^2 ^*= noise variance; *L_slp _*= 0.1 μm^-1^) caused a decrease in polarity of the no-positive-feedback model (NPF) in 1D simulations. The time-average of the polarity variable *a *(*ā*) with respect to *z *is depicted. The dashed gray line indicates response in the absence of input noise. **(C) **Spatial noise causes inaccurate polarization. In 2D simulations of the positive feedback model (PF), the directional accuracy was measured by comparing the response peak of polarization variable *a *to the direction of the input gradient. Increasing noise caused an inaccurate directional response. For each noise value, the left graph is a blue-to-red heat map representing the value of the variable *a*, and the right graph plots the same data as *a *versus the arc length *θ*. **(D) **Increasing the gradient slope (*L_slp_*) for a fixed noise strength (*σ *= 0.1) improved polarization in the NPF model. The extent of polarization (*ā *at front) increased with steeper gradients. The dashed gray line represents the polarization in the absence of input noise. **(E) **Plot of polarization versus signal-to-noise ratio. For simulations of the NPF model, the y-axis represents the *ā_f _*values normalized by subtracting 1. The *x*-axis represents the signal-to-noise ratio $\frac{L_{slp}}{\sigma }$ using a log-scale. Data is grouped into symbols according to *L_slp _*values as shown by the key.

A different challenge is faced by larger cells that use spatial rather than temporal information to orient to chemical gradients. Examples of such cells include hungry ameoba, patrolling neutrophils, swimming sperm, growing neurons, metastasizing tumor cells, and mating yeast. Spatial sensing entails measuring a difference in the concentration of an external cue between the front and back of the cell. Based on this information, the sensing cell decides whether or not to polarize in the direction of the gradient. Noise in the gradient, caused by Brownian motion, and convection, etc., can provide a substantial challenge to spatial sensing and response (Figure 1A).

Cell polarization refers to the behavior in which a cell responds to an internal or external cue [8,9] by localizing components that were previously uniformly distributed. One key aspect of polarization is the amplification needed to convert a shallow external gradient into a steeper internal gradient [10-12]; this allows the cell to respond decisively even to weak or shallow gradients. The danger is that the system may amplify noise instead of signal [13]. Three important properties of effective oriented polarization are tight localization (amplification), directional accuracy (tracking the gradient source), and noise-free output (smoothness) (Figure 1A).

Chemotaxis (moving towards or away from a gradient) involves gradient-sensing, polarization, and further mechanical events, such as the formation of lamellipodia at the leading edge. However, not all cells that sense gradients and respond by polarization are chemotactic. For example, haploid cells of the yeast *S. cerevisiae*, which are non-motile, can sense an external gradient of mating pheromone and grow a mating projection toward the source; this phenomenon is known as *chemotropism*. Thus, yeast cells represent pure spatial sensors: they do not move. As with chemotaxing cells, the initial response of yeast cells to a pheromone gradient is polarization, as evidenced by the large number of proteins that localize to the site of the incipient mating projection [14,15]. Much of the machinery that regulates this process is well-conserved in eukaryotes from yeast to humans, and includes both a heterotrimeric G-protein and the small G-protein Cdc42 [9,16].

Recently, Endres and Wingren [17] have extended the previous theoretical framework [5,6] to encompass spatial sensing. They calculated the ability of an immobile sphere to measure a chemical gradient. They showed good agreement between their theory and recent measurements of the gradient-sensing of *Dictyostelium *cells [2], which are motile. In the study of *Dictyostelium*, van Haastert and Postma [2] derived an expression for the signal-to-noise ratio (SNR) of sensing a chemical gradient in terms of the slope of the gradient, receptor affinity, integration time, and intracellular noise. In addition, Rappel and Levine [18] used Monte Carlo simulations to estimate receptor-ligand binding noise, a second important noise source, and then applied this noise input to two chemotaxis models. They demonstrated that this noise limited chemotactic efficiency. In a second paper, Fuller et al. [19] further developed an information theoretic measure of chemotactic performance [20] and compared experimental data using *Dictyostelium *cells to their theoretical bounds showing that at higher concentrations and gradient slopes chemotaxis performance was suboptimal. It is important to note that the above studies focused on chemotactic cells and measured performance by the chemotactic index.

There have been a number of recent results on budding, a yeast polarization behavior that is directed toward an internal cue, the bud scar. One focus has been on the positive feedback loops necessary for budding. Lew and colleagues have demonstrated the importance of the scaffold protein Bem1 [21,22] which participates in what has been termed the inner positive feedback loop [23]. Li, Altschuler and colleagues have investigated the role of actin-mediated endo/exocytosis directed by Cdc42 on polarized behavior [24,25], which represents a second outer positive feedback loop. In this paper, the emphasis is on the mating response and the polarization response to an external cue.

Another important branch of the mating pathway is the MAPK (mitogen-activated protein kinase) signaling system [26,27]. After G-protein activation, free Gβγ recruits the scaffold protein Ste5, which tethers the members of the MAPK cascade to the membrane. As a result, the MAPKs Fus3 and Kss1 become activated. They phosphorylate the trancription factor Ste12 triggering the mating transcriptional program inducing the production of morphology proteins such Fus1 [28], and the directed transport of mating proteins to the projection.

Here, we investigated how gradient noise affects spatial sensing and response; we used a generic model as a basis for numerical analysis, and then a yeast mechanistic model along with experiments to support our points. We focused on cell polarity and the challenge of amplifying a shallow, noisy input gradient to produce a tightly localized, directionally accurate, and relatively noise-free output polarization. We explored various noise filtering strategies and their tradeoffs in both gradient-sensing and the polarization response. We performed detailed simulations that characterized in a more quantitative fashion the balancing of the tradeoffs, and carried out microfluidics experiments that produced data consistent with the simulations. We concluded that yeast cells combine different noise filtering approaches resulting in a slow but accurate polarization response.

## Results

### Description of the generic model

Gradient-sensing and polarization response have been proposed to require the functions of several interacting modules [29]. These modules include a sensing module for gradient-sensing *per se*, an amplification module for amplifying a shallow external gradient into a steep internal gradient in which protein components are tightly localized at the front of the cell, and an adaptation module that allows the cell to sense and respond appropriately over a wide range of average concentrations of the external stimulus. We have previously developed a general, coarse-grained mathematical model of gradient-sensing and polarization response that allowed us to explore the interactions and tradeoffs among these modules, as well as to investigate the roles of ultrasensitivity, positive feedback, diffusion rates, multi-stage amplification, and other factors in the absence of noise [12]. Because of its abstract nature, the model applies to many different gradient-sensing scenarios, e.g. it applies just as well to neutrophil chemotaxis as it does to yeast mating. In this section we describe this model, and its extension to cover noisy inputs.

The equations for the general model are

(1-1) $$\frac{\partial a}{\partial t}=D_{s}\nabla_{s}^{2}a+\frac{k_{0}}{1+u^{-q}}+\frac{k_{1}}{1+{\left(pa\right)}^{-h}}-k_{2}a-k_{3}ba$$

(1-2) $$\frac{db}{dt}=k_{4}\left(ã-k_{ss}\right)b$$

$$ã=\frac{\int_{s}a\;ds}{\int_{s}ds}$$

$$p=\frac{1}{1+{\left(\beta u\right)}^{-q}}$$

The key variables in this model are *a *and *b*. Variable *a *represents the concentration of a membrane-bound protein whose polarization is a marker for the cell's response to the gradient. Variable *b *implements negative feedback regulation, as described further below. Equation 1-1 is written as a partial derivative because we are concerned with how *a *changes in both time and space. It consists of five terms that are added together. We will consider these terms in these equations individually. The first term describes the diffusion of *a*, with *D_s _*being the diffusion coefficient within the membrane.

The second term, which we will refer to as the 'input ultrasensitivity' term, describes the rate of change of *a *that depends on the external input signal *u*. The parameter *k*_0 _is a constant (i.e. fixed parameter) that determines the maximum value the input ultrasensitivity term can take. The fractional part of the term, which can be rewritten $\frac{u^{q}}{1+u^{q}}$, has a minimum value of 0 (which it will obtain when there is no input, when *u *= 0) and a maximal value of 1 (which it will approach, when the magnitude of the input is much greater than 1). The parameter *q *determines the cooperativity, or ultrasensitivity, of the response to input, with *q *= 1 indicating a non-cooperative, Michaelian, hyperbolic response, and *q *> 1 indicating a more sigmoidal, switch-like response. If *q *is large, then the input *u *need not be that much larger than 1 for the fractional part of the term to approach its maximum value of 1. One mechanistic interpretation of the input ultrasensitivity term is that the fractional component represents a saturatable ligand-receptor binding isotherm, and *k*_0 _represents the downstream signaling activity of ligand-bound receptors. However, the input ultrasensitivity term can also be interpreted more abstractly as a Hill function that describes the dose-response profile of an entire signaling cascade.

The third term in Equation 1-1 describes the positive feedback loop, with *a *positively regulating its own rate of change. This term will approach its maximum value of *k*_1 _when the product *pa *is much greater than 1, or when *pa *is greater than 1 and the Hill coefficient *h *is large. The expression *p *represents an input-dependent Hill term in the positive feedback loop; it assumes values between 0 and 1 depending upon the level of input. Because *p *is equal to zero when input *u *is zero, *p *prevents the positive feedback loop from "locking in" the absence of input. Previously [12], we showed that making the positive feedback input-dependent reduced the multi-stability at high positive feedback gains (i.e. getting stuck in one direction), thus improving the ability to track a gradient directional change.

The fourth term in Equation 1-1, -*k*_2_*a*, describes the simple first order decay of *a*, due to, for example, bulk protein degradation. Finally, the fifth term, -*k*_3_*ba*, describes the action of the global negative feedback regulator *b*, with parameter *k*_3 _specifying the strength of the feedback. A simple interpretation of this term is that when a molecule of *b *collides with a molecule of *a*, this may result in the inactivation of that molecule of *a*. The negative regulator *b *may be a specific protease, or a phosphatase that dephosphorylates and thereby inactivates *a*, etc. Unlike *a*, the spatial distribution of *b *does not respond to the gradient; in other words, the excitation is local and the inhibition is global. It is also important that the amount of *b *per cell is a variable that depends upon the difference between the current amount of *a *and a target level *k_ss _*(Equation 1-2); this is integral feedback control [12,27].

The parameter *ã *is the average value of *a *integrated over the cell surface and tends to *k_ss _*at steady-state because of the integral feedback. The integral feedback ensures robust adaptation to the level of input so that the total amount of *a *at steady-state is constant regardless of the input magnitude.

To summarize, the model contains two amplification terms that give rise to the polarization, as well as an integral negative feedback loop [30] to regulate the polarization. The two generic amplification mechanisms are an ultrasensitive dependence on the input *u *(*k_0 _*term), and a positive feedback loop (*k_1 _*term). With respect to the production of *a*, the parameters *k_0 _*and *k_1 _*modulate the balance between the input ultrasensitivity term and the positive feedback term. Both are formulated using a Hill expression; the Hill coefficients *q *and *h *were typically chosen in a range that allowed polarization in the absence of noise for a given gradient slope *L_slp _*[12]. When we performed parametric analysis on *k_0 _*and *k_1_*, we identified two distinct dynamical regimes: A low positive feedback regime corresponding to ultrasensitive amplification and a high positive feedback regime corresponding to positive feedback amplification (see Fig. S1, Additional file 1). Thus, we focused on two versions of the model: One in which ultrasensitivity was dominant and one in which positive feedback was dominant. The default value for the other *k_i _*parameters was 1; more model details are provided in Additional file 1.

In the second half of the paper, we relate the generic model to a more detailed mechanistic model of yeast cell polarity. In the generic simulations we examined some stereotypical situations. For example, we explored cases of extreme ultrasensitivity or extreme positive feedback. As a result, the values of some parameters were large, e.g. for high ultrasensitivity, the Hill coefficient *q *= 1000 was used. In practice, such a term could be implemented by a cascade of three reactions, each possessing a Hill cooperativity parameter of 10 [12].

We adopted a Langevin approach [1,31] to modeling external gradient noise: *u*(*x*, *t*) = *u'*(*x*, *t*) + *η*(*x*, *t*), where the input *u *is the sum of a deterministic static gradient input *u' *and a stochastic noise term *η*, with position denoted by *x *and time by *t*. We used either normal or log-normal white noise; the log-normal expression was adopted for high noise values to prevent negative input values (see Methods).

We employed both "one-dimensional" (axisymmetric) and "two-dimensional" (circle) simulations; the two produced equivalent results and were used interchangeably. The 1D simulations represented a sphere because of symmetry considerations and more readily depicted the extent of polarization along the axial direction. The 2D simulations were necessary for determining the polarization direction relative to the gradient direction when not correctly aligned. More complex geometries were beyond the scope of this paper.

### Input noise inhibits the extent, accuracy, and smoothness of polarization

We first examined how spatial gradient noise affected the extent of polarization (Figure 1A). This was measured by the value of *a *at the front of the cell (*a_f _*corresponds to the average value of *a *at the axial position *z *= 1 in Figure 1B). The integral feedback ensures that the spatial average *ã*~1, and thus greater amplification and tighter localization result in a larger *a_f_*. The magnitude of the noise was adjusted by changing its variance *σ*^2^. In the ultrasensitive model with no-positive-feedback (NPF, *k*_0 _= 10 and *k*_1 _= 0), we observed that there was a progressive loss of polarization as we increased *σ *from 0.01 to 1. At *σ *= 0.01, *a_f _*~ 2, whereas at *σ *= 1, polarization was nearly abolished, *a_f _*~ 1 (Figure 1B).

We next tested models with positive feedback (PF, *k*_0 _= 1 and *k*_1 _= 10) using 2D simulations in which the cell was represented as a circle. In this manner, we could assess directional accuracy by calculating the cosine of the angle *θ *of the polarity peak with respect to the gradient direction; cos(*θ*) is a typical index of mating projection directional accuracy [32] with cos(*θ*) = 1 indicating perfectly accurate polarization and cos(*θ*) = 0 indicating completely random polarization.

Indeed, the positive feedback models produced greater polarization than the NPF models. For each simulation, the polarization is depicted both on a circle color-coded for *a *values, and via a plot of *a *versus arc length. As before, increasing the noise resulted in a polarization decrease indicated by the shallower peak, but in addition, for *σ *= 1, the peak was not aligned with the gradient direction indicating a loss of polarization accuracy (Figure 1C). Thus, the directional accuracy of polarization was affected by the noise.

Third, we calculated the output noise in the simulations. In Figure 1B, using the NPF model (*L_slp _*= 0.1 μm^-1^), we found that increasing the input noise led to an increase in output noise as expected. Figure 1B depicts the spatial variations in *a*. There was also an increase in the temporal variance, which we measured as the standard deviation *σ_out _*in *a_f _*as a function of the input noise magnitude for four *σ *values: (*σ*, *σ_out_*) = (0.01, 0.001), (0.1, 0.1), (1.0, 0.18), (10, 0.23). Thus not surprisingly, increasing the input noise led to an increase in output noise.

It was not the magnitude of the noise alone that mattered, but the magnitude of the noise with respect to the slope of the gradient. We explored three values of the slope in the NPF model and monitored the extent of polarization for a fixed noise value (Figure 1D). Polarization increased as we increased the slope. For a given ratio of gradient slope to noise magnitude the extent of polarization was roughly equivalent (Figure 1E, Table S2 in Additional file 1). This result supports the gradient slope dependence of the signal-to-noise ratio (SNR) derived by van Haastert and Postma [2] and others [17,19,33]. In addition, there was approximately a linear relationship between *a_f _*and log(SNR). However, it should be noted that the equivalence deviated at larger slope values (e.g. *L_slp _*= 0.3 μm^-1^), which produced higher than expected polarization values. One interpretation is that the effect of noise on polarization was reduced because less amplification was needed to convert a steep external gradient into the internal polarization.

Finally, we addressed the question of how the quality of polarization varied as we changed the amplification in the models. In the NPF model, increasing the Hill coefficient *q *resulted in larger values of *a_f_*, but bigger *q *also increased the output noise, i.e., both signal and noise were amplified (Table S3, Additional file 1). In the PF model, increasing the positive feedback gain via the parameter *h*, resulted in an increase in the polarization extent *a_f_*, but reduced the directional accuracy cos(*θ*) (Table S3, Additional file 1). Thus, varying a parameter can improve one aspect of polarization but detrimentally affect another. Ultimately, one would like to combine the various elements of polarization - extent, accuracy and smoothness - to derive a single measure of polarization quality.

### Noise filtering strategies

**Figure 2:**
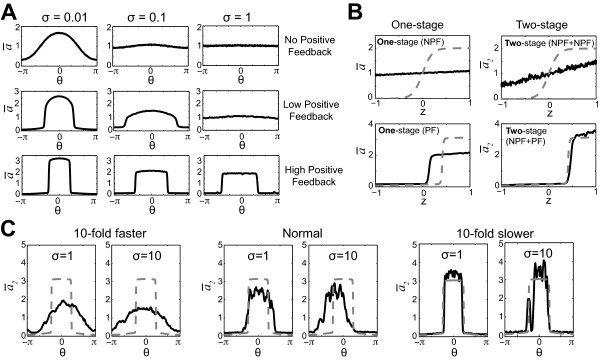
**Exploring noise-filtering strategies**. **(A) **Increasing the positive feedback improved the extent of polarization at higher noise levels. 2D simulations were performed on the model in which the strength of the positive feedback ranged from none (*k_1 _*= 0) to low (*k_1 _*= 10, *h *= 2) to high (*k_1 _*= 10, *h *= 8). For a given gradient (*L_mid _*= 1, *L_slp _*= 0.01 μm^-1^), higher positive feedback produced stronger polarization at higher levels of noise. The time-average of the polarity variable *a*(*ā*) with respect to *θ *is plotted. **(B) **Adding a filtering module improved polarization. In the top row, there was a single NPF (no-positive-feedback) module compared to two NPF modules in series. In the second row, there was a single PF (positive feedback) module compared to a two-stage arrangement of a NPF module followed by a PF module (NPF+PF). The extra stage resulted in improved polarization. Dashed gray lines represent polarization in the absence of input noise (1D simulations with *L_slp _*= 0.01 μm^-1^, *σ *= 0.1). **(C) **Slower dynamics produced more accurate and effective polarization. In a two-stage NPF+PF model, we scaled the parameters either 10-fold faster or 10-fold slower in 2D simulations (*L_slp _*= 0.01 μm^-1^). We plotted the spatial distribution of the output of the second stage *ā*_2 _for two values of *σ*.

It is critical for a cell to operate robustly in a noisy environment. In this section we explored filtering strategies for attenuating noise during cell polarization. Previous work focused on noise reduction in non-spatial systems [13,23,34,35], whereas we focused on filtering spatial input noise for a spatial response. Positive feedback can create switch-like behavior in both non-spatial and spatial systems, and this bistability can make a system more robust to noise by locking the output to a particular value [36,37]. Here we tested the effect of positive feedback on our model of cell polarization. The gain of the positive feedback was adjusted through the parameters *k_1 _*and *h *in the positive feedback term. In simulations with *L_slp _*= 0.01 μm^-1^, and for *σ *= 0.1, 1, 10, we observed that increasing the positive feedback gain resulted in enhanced polarization at higher noise values (Figure 2A).

As noted in the previous section, higher positive feedback at large noise values can reduce directional accuracy, and we wished to combine polarization extent and direction into a single measure to assess more quantitatively the benefits of positive feedback. One possible measure is to normalize *a_f _*to 0 by subtracting *k_ss _*(the value of *ã *at steady-state = 1), and then to multiply this number by cos(*θ*) to represent the component in the direction of the gradient: $a_{f}^{norm}=\left(a_{f}-1\right)\cos\left(\theta \right)$; the larger this value indicates better polarization in the correct direction. Using this measure for one set of input conditions (Table S3, Additional file 1), we observed that high positive feedback (*h *= 8, $a_{f}^{norm}=0.81$) did indeed significantly outperform low positive feedback (*h *= 2, $a_{f}^{norm}=0.07$).

A second strategy is to use multiple stages in the signaling pathway in which an early part of the pathway (e.g. first stage) acts as a filter for a later part of the pathway (e.g. second stage) [38]; Iglesias noted that this architecture is common in technological systems. We tested an arrangement in which the no-positive-feedback (NPF) module acts as a filter for a second stage positive feedback (PF) module, which is responsible for the amplification. We denoted this architecture as a filter-amplifier. We observed that the noise decreased after the filtering as a function of the integration time of the module [39]. As a result, the two-stage models were able to handle the noise better than the single stage models with the same parameters (Figure 2B), showing greater polarization extent.

Time-averaging is perhaps the best known approach to filtering noise [13,34,39,40]. Using the two-stage model, we scaled the rate constants 10-fold larger (faster) or smaller (slower). At the slower speeds, there was better and more accurate polarization (Figure 2C). Two factors contributed to this effect. First, the noise after the first-stage showed the expected reduction in noise variance from slower averaging [13]. In addition, the positive feedback in the second stage was more effective and accurate at the slower speeds. As a result, there was a significant combined effect.

Finally, we explored whether increasing the surface diffusion could "smooth" the output. Indeed, for higher diffusion values, the response was less noisy. However, there was a cost in terms of reduced polarization (Fig. S2, Additional file 1).

### Tradeoff between noise filtering and response speed

Each noise-filtering strategy improved the accuracy and extent of polarization in the presence of spatial noise, but at what cost? From systems theory one expects a tradeoff with the speed of the system because a slower frequency response profile would cutoff the higher frequency noise [41,42]. We wished to examine this tradeoff in greater detail.

To assess response speed, we performed simulations in which we applied the gradient without noise and measured the time for the output to settle to within 5% of the steady-state value, and then we switched the gradient direction and measured the settling time for the directional change. A slower response resulted in a longer settling time *t_s _*after both the initial and switched gradient.

**Figure 3:**
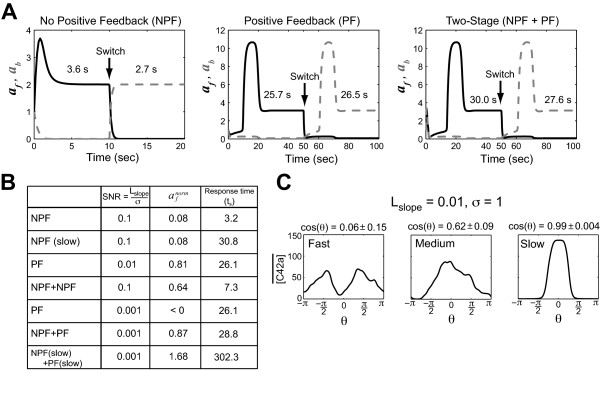
**Tradeoff between polarization and response time**. **(A) **The black solid line describes time evolution of *a_f_*, value of *a *at the front; the gray dashed line represents the evolution of *a_b_*, value of *a *at the back. We measured the response time to an initial gradient and then switched the gradient direction. Response time is defined as the time it takes the simulation to settle to within 95% of the steady-state value. The initial gradient was applied and then after reaching steady-state, the gradient direction was switched 180^o ^(*L_mid _*= 1 and *L_slp _*= 0.01 μm^-1^). Results for the NPF (left), PF (middle), and NPF+PF (right) models are shown along with the response times. The calculated settling time *t_s _*is the average of the initial and switched response times. **(B) **A comparison of different model architectures in terms of polarization $\left(a_{f}^{norm}\right)$ and response time (*t_s_*). For the NPF models, a larger signal-to-noise (SNR) was needed to observe polarization. In the slow versions of the models, the parameters were scaled 10-fold lower. Two-stage models are indicated by the "+" between the stages. **(C) **Simulations of yeast model at different speeds for the heterotrimeric G-protein cycle. The speed was normal (wild-type parameter values), 10-fold faster, and 10-fold slower. At least 20 simulations were performed and the mean value of active Cdc42 in the simulations are plotted against the angle of the cell (*θ *= 0 is the direction of the gradient). The mean ± SEM of cos(*θ*) is shown above the plot.

Indeed, each of the noise-filtering strategies resulted in a slower response time (Figure 3A). Time-averaging is achieved by slowing the system down which will slow the response. The positive feedback system (PF, *t_s _*= 26.1 s) was slower than the system without positive feedback system (NPF, *t_s _*= 3.2 s). Adding an additional stage to create a two-module cascade resulted in a summation of the response times. Interestingly, adding an NPF module in front of a PF module resulted in a big improvement in polarization in the presence of spatial input noise (Figure 2B) with the cost of a relatively modest increase in the response time (NPF + PF, *t_s _*= 28.8 s) compared to PF alone.

We then explored these tradeoffs in greater quantitative detail. We investigated different speeds for the models, compared two stages versus one stage, and examined the filter-amplifier structure of the NPF+PF model. A common feature of the noise-filtering strategies was that an improvement in polarization resulted in a slower system response, although the nature of this tradeoff varied. The positive feedback model exhibited superior polarization but was slower than the no positive feedback model containing similar parameter values (Figure 3B, NPF versus PF); a two-stage system performed better but was slower than a one-stage system (Figure 3B, NPF versus NPF+NPF or PF versus NPF+PF); and taking a model and scaling the parameters to a slower speed resulted in much improved polarization (Figure 3B, NPF+PF versus NPF(slow)+PF(slow)). From these simulations, we observed that the best combination was the NPF filter followed by the positive feedback because the filter did not add too much time delay while removing a significant amount of noise. As a result, the NPF+PF filter-amplifier model produced good polarization at low SNR with a response time in the same range as the single-stage model structures.

### Modeling the yeast polarization response to gradient noise

We previously [11,12] constructed a spatial model of yeast cell polarization (Additional file 1). Here, we tested the ability of this model to filter input noise and polarize in the correct direction. The model contains many features described for the generic model that would help filter noise. First, the system consists of two stages: The heterotrimeric G-protein cycle represents the first stage which then feeds into the Cdc42 cycle, the second stage. Second, there are two positive feedback loops, an inner fast loop involving the scaffold protein Bem1 and the small G-protein Cdc42, and an outer slower loop involving polarized synthesis of receptor [23,24]. Finally, the system is slow compared to other G-protein systems such as visual phototransduction [43]. The model is presented below:

(2-1) $$\begin{array}{c} \frac{\partial \left[\text{R}\right]}{\partial t}=D\nabla_{s}^{2}\left[\text{R}\right]-k_{RL}\left[\text{L}\right]\left[\text{R}\right]+k_{RLm}\left[\text{RL}\right]\\ -k_{Rd0}\left[\text{R}\right]+p_{s}k_{Rs} \end{array}$$

(2-2) $$\frac{\partial \left[\text{RL}\right]}{\partial t}=D\nabla_{s}^{2}\left[\text{RL}\right]+k_{RL}\left[\text{L}\right]\left[\text{R}\right]-k_{RLm}\left[\text{RL}\right]-k_{Rd1}\left[\text{RL}\right]$$

(2-3) $$\frac{\partial \left[\text{G}\right]}{\partial t}=D\nabla_{s}^{2}\left[\text{G}\right]-k_{Ga}\left[\text{RL}\right]\left[\text{G}\right]+k_{G1}\left[\text{Gd}\right]\left[\text{Gbg}\right]$$

(2-4) $$\frac{\partial \left[\text{Ga}\right]}{\partial t}=D\nabla_{s}^{2}\left[\text{Ga}\right]+k_{Ga}\left[\text{RL}\right]\left[\text{G}\right]-k_{Gd}\left[\text{Ga}\right]$$

(2-5) $$\frac{\partial \left[\text{Gbg}\right]}{\partial t}=D\nabla_{s}^{2}\left[\text{Gbg}\right]+k_{Ga}\left[\text{RL}\right]\left[\text{G}\right]-k_{G1}\left[\text{Gd}\right]\left[\text{Gbg}\right]$$

(2-6) $$\frac{\partial \left[\text{Gd}\right]}{\partial t}=D\nabla_{s}^{2}\left[\text{Gd}\right]+k_{Gd}\left[\text{Ga}\right]-k_{G1}\left[\text{Gd}\right][\text{Gbg]}$$

(2-7) $$\begin{array}{ll} \frac{\partial \left[\text{C}24\text{m}\right]}{dt} & =D\nabla_{s}^{2}\left[\text{C}24\text{m}\right]+k_{24cm0}\left({\text{Gbg}}_{\text{n}}^{*}\right)\left[\text{C}24\text{c}\right]\;\\ & +\;k_{24cm1}\left(\text{B}1^{*}\right)\left[\text{C}24\text{c}\right]-k_{24mc}\left[\text{C}24\text{m}\right]\;\\ & -\;k_{24d}\left[\text{Cla}4\text{a}\right]\left[\text{C}24\text{m}\right]\;\\ & \; \end{array}$$

(2-8) $$\frac{\partial \left[\text{C}42\right]}{dt}=D\nabla_{s}^{2}\left[C42\right]-k_{42a}\left[\text{C}24\text{m}\right]\left[\text{C}42\right]+k_{42d}\left[\text{C}42\text{a}\right]$$

(2-9) $$\frac{\partial \left[\text{C}42\text{a}\right]}{dt}=D\nabla_{s}^{2}\left[\text{C}42\text{a}\right]+k_{42a}\left[\text{C}24\text{m}\right]\left[\text{C}42\right]-k_{42d}\left[\text{C}42\text{a}\right]$$

(2-10) $$\frac{\partial \left[\text{B}1\text{m}\right]}{dt}=D\nabla_{s}^{2}\left[\text{B}1\text{m}\right]+k_{B1cm}\left[\text{C}42\text{a}\right]\left[\text{B}1\text{c}\right]-k_{B1mc}\left[\text{B}1\text{m}\right]$$

(2-11) $$\frac{\text{d}\left[\text{C}1\text{a}4\text{a}\right]}{dt}=k_{Cla4a}\left(C42a_{t}^{*}\right)-k_{Cla4d}\left[\text{Cla}4\text{a}\right]$$

Equations 2-1 to 2-6 represent the heterotrimeric G-protein cycle (first stage). Receptor (R) binds ligand (L) to form the activated receptor-ligand complex (RL). This complex catalyzes the production of active G-protein, both Gα-GTP (Ga) and free Gβγ (Gbg). Equations 2-7 to 2-11 represent the Cdc42 cycle (second stage). The inner positive feedback loop is mediated by Bem1-Cdc24-Cdc42 in Equations 2-7 to 2-10: Cdc24 is the activator of Cdc42, and active Cdc42 (C42a) binds the scaffold protein Bem1 on the membrane (B1m), which recruits more Cdc24 (C24m) to a particular location. There is a second slower outer positive feedback loop in which active Cdc42 organizes the actin cytoskeleton to direct transport of new proteins to the mating projection. The key protein affected by this polarized transport is receptor which is endocytosed and exocytosed during the pheromone treatment. Thus, we represented the outer positive feedback loop by the polarized synthesis term *p_s_*, which depends on the fractional activation of Cdc42 (i.e. active Cdc42 at position *x *over the spatial average of active Cdc42) at different positions on the membrane:

$$p_{s}=\frac{[\text{C42a]}}{C42a_{t}^{*}},\text{if}\;C\text{42}a_{t}^{*}>0,\text{else}\;p_{s}=1$$

More detailed justifications for the model equations and parameters are provided in Additional file 1.

We wished to estimate the noise variance *σ^2 ^*for the ligand α-factor in simulations of this model. Yeast cells contain approximately 10,000 receptors evenly distributed over the surface of a sphere with radius 2 μm [44]. In the simulations, each grid point at which the noise was applied represented approximately the neighborhood around a single receptor. From the theory [6], we obtained an estimate of the noise level in our simulations of the yeast system to be *σ *~ 1.3 to 4.1 (Additional file 1). Although we primarily addressed external ligand-diffusion noise, in Additional file 1, we investigated the combined effect of ligand-diffusion noise and receptor-ligand binding noise (Table S4). The latter contributes significantly to variations in the level of receptor-ligand complex [18,33,45]. In this paper the goal was to focus on "external" noise and the limitations imposed by this noise.

As demonstrated in the previous section, we would expect that a filtering stage would improve polarization and that the filtering is better at slower speeds. We tested the model at a 10-fold faster speed and a 10-fold slower speed for the heterotrimeric G-protein cycle with a gradient slope of *L_slp _*= 0.01 nM μm^-1 ^(*L_mid _*= 10 nM, concentration at cell midpoint) and *σ *= 1. Directional accuracy in 2D simulations was assessed by calculating cos(*θ*), *θ *= angle between the gradient and polarization direction. In Figure 3C, slowing the speed of the heterotrimeric G-protein portion of the model improved projection accuracy from cos(*θ*) = 0.06 (fast) to 0.62 (normal) to 0.99 (slow). Thus, we demonstrated how slowing the first stage creates a better filter for the second stage amplifier resulting in more accurate polarization.

### Both positive feedback loops are necessary for noise-resistant polarization

From the simulations of the generic model, we expect that positive feedback loops are necessary for potent polarization in the presence of noise. The mechanistic yeast model described above contains not one, but two positive feedback loops, a slow outer and a fast inner loop. Interlinked fast and slow positive feedback loops can be beneficial for achieving both rapid induction of a response and noise resistance [23]. One example was the yeast budding behavior in which the Bem1 positive feedback loop was fast and the Cdc42 actin loop was slow. These simulations were non-spatial, and here we explored spatial simulations in the context of cell polarity directed by an external cue (spatial gradient). As noted in the Background section, both positive feedback loops have been characterized individually with respect to budding [22,24], but their relative roles and how they interact in the context of pheromone-induced polarization have not been explored in depth.

**Figure 4:**
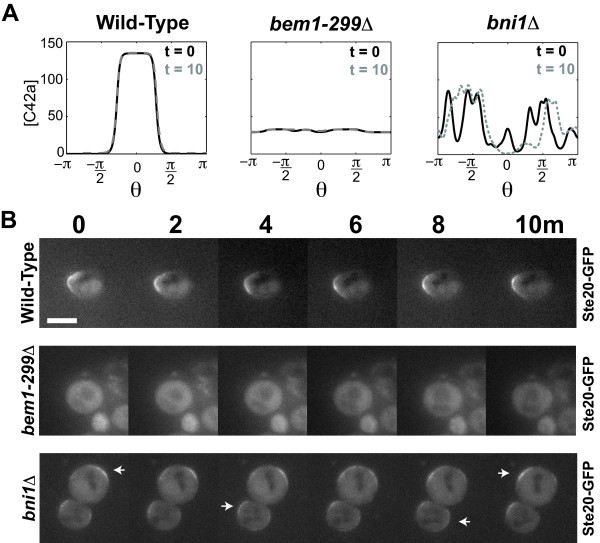
**Both the inner and outer positive feedback loops are necessary for noise-tolerant polarization**. **(A) **Time-course simulations of wild-type, *bem1-299Δ*, and *bni1Δ *models. After approaching steady-state, the models were run for 10 minutes of simulated time (*L_mid _*= 10 nM, *L_slp _*= 0, *σ *= 3). For a sample simulation, active Cdc42 is plotted against the angle *θ *for *t *= 0 (solid black) and *t *= 10 min (dashed gray). **(B) **Time-lapse imaging of wild-type, *bem1-299Δ*, and *bni1Δ *cells containing Ste20-GFP, a marker of active Cdc42. Cells were treated with uniform 20 nM α-factor for 1 hour and imaged every 2 min for 10 minutes. 20 cells of each genotype were observed and a typical cell is shown. All wild-type cells showed a constant region of polarization, whereas all *bem1-299Δ *cells did not polarize with the Ste20-GFP mainly cytoplasmic. In the majority *bni1Δ *cells (14/20), there were multiple regions of polarization (indicated by white arrows) that shifted position over the 10 minute interval; some cells exhibited only cytoplasmic Ste20-GFP. Scale bar = 5 μm.

We ran simulations in which the inner loop or the outer loop was disrupted, and monitored the spatial dynamics of active Cdc42 (C42a). First, we ran simulations in which we removed the inner loop by setting *h *= 0 for the Bem1 positive feedback term; there was a total loss of polarization (Figure 4A, middle) with lower levels of active Cdc42 on the membrane. Then, we ran simulations in which the outer loop was eliminated by setting the polarized synthesis of receptor to 0 (*p_s _*= 0). In these simulations we observed multiple polarization peaks around the cell that shifted position over the course of 10 minutes (Figure 4A, right). In contrast, the wild-type simulations displayed a single stable polarization peak (Figure 4A, left).

Experimentally, we monitored the spatial dynamics of active Cdc42 using Ste20-GFP as a reporter [46] over a 10 minute time period. In wild-type cells, Ste20-GFP localized to the front of the projection in a stable fashion (Figure 4B, top). In a *bem1-299Δ *mutant [47], a C-terminal truncation of Bem1 that does not bind Cdc24 breaking the Cdc24-Cdc42-Bem1 positive feedback loop, most Ste20-GFP was cytoplasmic indicating reduced Cdc42 activity; the lack of polarization on the membrane was in agreement with simulations. Then we examined a *bni1Δ *mutant [48]; Bni1 is a formin [14] that makes the actin cables necessary for polarized synthesis and transport of proteins such as receptor to the mating projection during the pheromone response. These spatial dynamics are central to the outer positive feedback loop in which polarized endocytosis and exocytosis localizes cell components to the front. In the absence of Bni1, newly synthesized protein is transported in an approximately isotropic fashion via the other yeast formin Bnr1. Interestingly, *bni1Δ *cells exhibited a dynamic polarization region that shifted position over time (Figure 4B, bottom; Video S1, Additional file 2) as observed qualitatively in the simulations.

These results can be understood in terms of an initial polarization induced by the inner Cdc42 positive feedback loop that was reinforced by the slower outer positive feedback loop. Indeed in the simulations, after the initial polarization we observed a more gradual polarization of receptor and activated G-protein, which represented a slower amplification of the first stage heterotrimeric G-protein cycle. Without this reinforcement, the initial polarization was subject to disruption by the noise and was unstable. This view is consistent with the timing observed experimentally in which one observes active Cdc42 polarization, which is followed by receptor and G-protein polarization (Yi *et al*., data not shown).

### Shallowest gradient detected by yeast cells

In the absence of noise, the system is able to respond to shallow gradients by increasing the amplification. In the generic model, adjusting the positive feedback gain can result in infinite amplification [10,12,49]. However, the ability to sense and respond in the proper direction depends on the signal-to-noise ratio. Thus, we used our yeast model to predict the shallowest slope that could result in directional sensing given the estimated amount of ligand noise, and also to explore the extent directional sensing was limited by this noise.

**Figure 5:**
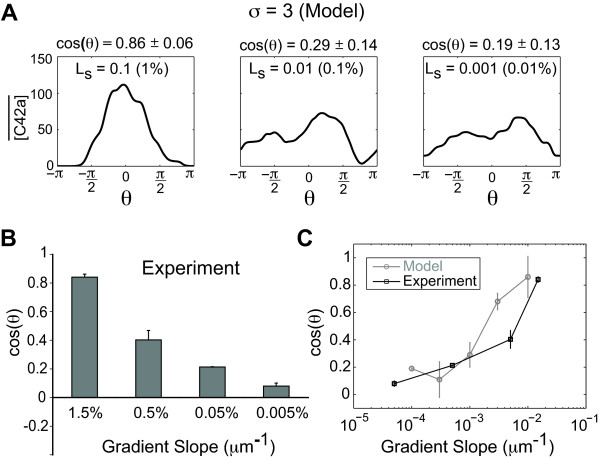
**Directional accuracy in yeast model simulations and microfluidics experiments**. **(A) **Simulations of yeast model at different gradient slopes. *L_slp _*= 0.1, 0.01, and 0.001 nM μm^-1 ^(*L_mid _*= 10 nM) and *σ *= 3. Using the wild-type model, at least 20 Monte Carlo simulations were performed as described above. The mean value of active Cdc42 is plotted, and the mean ± SEM of cos(*θ*) is also shown for each slope. **(B) **Directional projection accuracy at different gradient slopes. Experiments were performed for 1.5% gradient, 0.5%, 0.05%, and 0.005% μm^-1 ^gradients (*L_slp_*/*L_mid_*). Cells were counted in the middle two sections of the gradient where *L_mid _*~ 20 nM. Directional accuracy was measured in terms of cos(*θ*), and the mean ± SEM is shown for *n *= 3 trials. **(C) **Plotting yeast mating projection directional accuracy as a function of gradient slope for both experiments and modeling. Simulation data (gray) is from (A) and experimental data (black) is from (B).

We performed Monte Carlo simulations of the yeast model at normal speed using different gradient slopes (*L_slp _*= 0.1, 0.01, and 0.001 nM μm^-1^, *L_mid _*= 10 nM) and *σ *= 3 (Figure 5A). The results of the simulations showed that the model was able to sense the gradient of 0.1% (*L_slp_/L_mid_*) μm^-1 ^with cos(*θ*) = 0.29 ± 0.14. For the shallowest gradient (0.01% μm^-1^), the gradient sensing and response was closer to random, cos(*θ*) = 0.19 ± 0.13.

We compared computer simulations to experiments by examining projection direction of cells exposed to microfluidically-generated gradients [50-52]. To maximize the number of responding cells we set *L_mid _*= 20 nM in the experiments. We subjected cells to four gradients: 1.5% μm^-1^, 0.5% μm^-1^, 0.05% μm^-1^, and 0.005% μm^-1^. In Figure 5B, we observed that cells were able to sense and respond to the 0.05% gradient (cos(*θ*) = 0.21 ± 0.002) in agreement with the simulations and matching the ability of motile eukaryotic cells like *Dictyostelium *[2]. Interestingly, cells exhibited a directional response even in the 0.005% gradient (cos(*θ*) = 0.08 ± 0.01), but it was less accurate and closer to random than the response in the 0.05% gradient. For the steepest gradient (1.5% μm^-1^), the accuracy was good (cos(*θ*) = 0.8) as expected. In addition to enabling higher signal-to-noise, a very steep gradient slope could produce a saturation response that made the system less sensitive to noise. Overall, there was good agreement between experiments and modeling (Figure 5C). The shallowest gradient represents a single receptor-ligand molecule difference between front and back, and this calculation is consistent with previous estimates based on yeast gradient-sensing at high concentrations of α-factor [52]. Thus, the experiments and simulations suggest that external noise is likely to be a limiting factor in the ability of yeast cells to detect and respond to shallow spatial gradients in a directionally accurate fashion.

## Discussion

In this work, we performed mathematical modeling to investigate the effects of input spatial noise on cell polarization. Using a generic model of cell polarity, we demonstrated that input noise impeded the extent, accuracy, and smoothness of polarization. A key determinant was the ratio of the gradient slope (signal) to the noise magnitude. Different modes of amplification (ultrasensitive NPF versus positive feedback) exhibited different sensitivities to noise. For example, in the NPF models, it was possible to obtain accurate sensing but poor polarization amplification, whereas models possessing strong positive feedback produced potent polarization but inaccurate directional sensing under high noise conditions.

We explored noise-filtering strategies including time-averaging, multiple stages, and positive feedback, and described the tradeoff between filtering and response time. In the presence of noise, spatial amplification by positive feedback produced better polarization than amplification by an ultrasensitive mechanism. A good strategy was having a filter stage followed by a positive feedback stage, both possessing slow spatial dynamics. Our model of yeast cell polarity possessed this structure with the heterotrimeric G-protein cycle acting as the filter and the Cdc42 system acting as the amplifier resulting in a slow two-module filter-amplifier architecture [38]. In the future, it will be important to explore more input conditions, parameter values, and model structures by simulation, as well as develop the theory explaining the nature of the tradeoffs, and define hard constraints.

This work extends previous research on noise affecting cellular chemotaxis [2,5,6,17], but in the context of a pure spatial sensor that does not move. Cell polarization and the amplification of a shallow external gradient to a steeper internal gradient apply to both motile and immotile cells. As we note (Additional file 1), there is a close relationship between the chemotactic index and the cos(*θ*) measure of polarity. In the future, we would like to combine the different aspects of cell polarity - extent, direction, and smoothness - into a single measure.

We examined the minimum gradient slope that could be sensed by a spatial sensor in the presence of noise and produce a directional polarization response. We demonstrated that ligand noise places a severe limitation on the accuracy of the cell projection. Importantly, the key statistic was the ratio between the gradient slope (signal) and the noise variance. Thus, the noise places a limit on how shallow of a gradient a cell can sense and respond to accurately. Indeed, we found that wild-type yeast cells could project, albeit with imperfect accuracy, in a 0.1% μm^-1 ^gradient, which is close to the limit determined from the modeling. This value compares favorably to the chemical gradients that other chemotactic systems such as *Dictyostelium *[2] and neurons [33] can sense, and corresponds roughly to sensing a single receptor-ligand molecule difference front versus back.

The ability to sense the direction of gradients as shallow as 0.1% μm^-1 ^represents a challenging behavior. An important physiological requirement, which is not explicitly modeled in this paper, is that the magnitude of internal noise is small relative to external ligand noise, so that internal stochasticity does not interfere with gradient-sensing. One expectation is that there are large numbers of proteins in the system to minimize random reaction fluctuations. Indeed that is the case for the heterotrimeric G-protein cycle; there are approximately 10,000 receptors/cell as well as 10,000 G-protein subunits per cell [44].

From an evolutionary perspective, this extremely sensitive gradient-sensing may be useful when mating partners are separated by long distances. For example, assuming a point source emitting a 1 μM concentration of α-factor at a rate of 1 × 10^-8 ^ml/s (from a micropipette or clump of cells), the concentration at 1 mm would be ~10 nM with a slope of ~0.1% μm^-1 ^[32]. Although an individual cell could not project this distance to a mating partner, the cell could undergo pheromone-gradient directed filamentous growth [53] so that eventually one of its offspring could reach the source.

The work of Brandman et al. [23] highlighted the role of interlocked positive feedback loops on noise attenuation. Here we examined the implications in a spatial model, and the simulations showed that both feedback loops were necessary for proper polarization with the inner loop acting as the primary amplifier and the outer loop acting to maintain the polarization in a single location on a slower time-scale. Thus, the basic message in the spatial setting with interlocked positive feedback loops is that they reinforce each other to achieve a noise-tolerant polarization response.

Our results suggest the yeast cells have been optimized for cell polarity in spatial gradients. From the modeling we expect that a pure spatial sensor like yeast should polarize slowly in order to filter external noise, and we predict mutants that polarize faster should be less accurate. Conversely, one expects that at slower time scales the accuracy would be improved, and indeed, projection accuracy improves over time [52].

In this research we did not include internal noise in the simulations. The goal was to focus on the constraints placed by the external gradient noise. In the future, we plan to explore the impact on cell polarity of receptor-ligand binding noise, as well as internal signaling noise. For the yeast model, we estimated that the combination of external ligand noise and receptor-ligand binding noise, which is expected to increase the total noise on receptor-ligand (RL) levels, was still within the magnitude of noise values studied here. It would be interesting to compare the magnitude of the internal noise versus the external noise, and the problems as well as benefits created by this internal stochasticity on cell polarity.

Finally, we would like to add improvements to the yeast model for future research. In particular, it is important to include missing dynamics such as MAPK signaling [51,54,55]. It will be interesting to explore integrating a spatial model of the MAPK pathway [56] with the spatial dynamics of G-protein and Cdc42 signaling in the current model.

## Conclusions

Below we outline the main conclusions of this paper:

Noise in the input spatial gradient inhibits three aspects of cell polarization: Extent, directional accuracy, and smoothness. The signal-to-noise ratio can be represented as the slope of the gradient to the magnitude of the noise. This research represents the first attempt to use simulations in a generic fashion to dissect these effects more quantitatively.

There are three basic noise-filtering strategies: Positive feedback, multiple stages, and time-averaging. We explored the magnitude of their benefits individually and in combination. A systematic analysis of noise-filtering strategies has not previously been applied to the cell polarity problem.

There was a fundamental tradeoff between noise-filtering and the speed of the polarization response. A filter-amplifier structure in which an NPF stage is followed by a PF stage did the best job of balancing this tradeoff. In the context of cell polarity, we used simulations to explore this tradeoff with respect to different architectures, which was novel.

Our mechanistic model of yeast pheromone-induced cell polarity revealed this filter-amplifier structure with the heterotrimeric G-protein cycle system representing the filter stage and the Cdc42 cycle representing the amplifier stage.

The yeast system contains two positive feedback loops and both are essential for robust polarization in the presence of noise. Through simulations and experiments we demonstrated that the inner loop (Cdc24-Cdc42-Bem1) represents the principal amplifier stage. The slow outer loop helps to maintain the polarization in a single location by slow amplification of the first stage. Thus, the two interlocking positive feedback loops create a special dynamic that was not explored in previous work investigating non-spatial models of the system.

Finally, we showed how noise places limits on the directional accuracy of polarization. Microfluidics experiments demonstrated that yeast cells are able to sense the direction of very shallow gradients albeit imperfectly. The observed limits are close to those predicted from simulations of our model. The single receptor-ligand molecule spatial sensitivity is comparable to that of other eukaryotic gradient-sensing systems.

## Methods

### PDE simulations and noise model

The external noise is assumed to be spatially uncorrelated, and so noise at each spatial point was generated by selecting independent and identically distributed stationary random variables at a given time step. We used either a normal or log-normal distribution with mean 0 and standard deviation *σ*. The log-normal distribution was used when *σ *was large compared to *L_mid _*to prevent negative input values. The two distributions produced approximately the same effect on polarization on control simulations of the NPF model.

Because of computational discretization, noise was added at each grid point on the cell surface at a specified time interval. We explored a range of spatial resolutions (*k_x _*= 40 to 200 grid points) and temporal resolutions (noise time step *k_t _*= 0.001 to 1 s) and found only minor differences in control simulations. We chose as the default values *k_x _*= 200 and *k_t _*= 0.01 s.

The solutions were typically observed at a time long enough ("steady-state") for all the variables at each spatial point to reach an approximately invariant distribution. To estimate the stationary distribution from one simulation, 10000 samples were taken over a time interval of 100 s at each spatial point, and the mean and standard deviation were calculated.

### Mathematical models

The generic models and yeast mechanistic models were derived from Chou et al. [12]. More details are provided in Additional file 1.

### Monte Carlo estimation of directional accuracy

To estimate statistically how the system behaves under noise with respect to directional accuracy, we performed Monte Carlo simulations, which is a repeated computation of the stochastic models. In this paper, the number of the samplings in the Monte Carlo simulations ranged from 20 to 40. Each sampling simulation was run until the system reached an invariant distribution, and the mean and variance were calculated over the solutions. The polarization direction was calculated from the center of mass of the polarized species (active Cdc42).

### Strains

Standard methods for yeast genetic and molecular biology techniques were performed [57]. The strain genotypes are listed in Table S1. Yeast cells were cultured in rich YPD media supplemented with adenine (YPAD).

### Time-lapse imaging

To observe single cells, exponentially growing cells were treated with 20 nM α-factor for 1 hour and then imaged live every minute on concanavalin A treated slides for 10 min. The prepared slides were observed using a Nikon ECLIPSE TE300 inverted microscope.

### Microfluidics

As previously described, we used a standard Y-chamber microfluidics device to generate the α-factor spatial gradients [52]. The device was 800 μm in width, which was divided into 8 regions. The cells were subjected to α-factor gradients for 5 h, and for the projection directional accuracy measurements, cells located in the middle regions 4 and 5 were assessed (*L_mid _*= 0.5·(*L*_max _+ *L*_min_), *L_slp _*= 0.0025·(*L*_max _- *L*_min_) μm^-1^).

## Authors' contributions

CSC carried out all computer simulations, helped conceive and plan the research, analyzed the data, and contributed to the writing of the draft. QN helped conceive and plan the research, analyzed the data, and edited the manuscript. LB wrote several sections of the draft, and edited the manuscript. TMY carried out all experiments, helped conceive and plan the research, analyzed the data, and wrote most of the draft, and edited the manuscript. All authors read and approved the final manuscript.

## Supplementary Material

Additional file 1 **Supplemental Material**. This file contains Table S1 (Yeast strains), a description of the mathematical models, Figure S1 (Parametric analysis of *k_0 _*and *k_1_*), Table S2 (Effect of gradient slope versus noise on polarization), Table S3 (Effects of noise on polarization quality), a section estimating external gradient noise, Table S4 (Effects of ligand diffusion noise (*σ_L_*) and receptor-ligand binding noise (*σ_RL_*) on projection directional accuracy), a comparison of chemotactic index to cos(*θ*) measure of directional accuracy, Table S5 (Effect of diffusion of the polarized species on polarization), Figure S2 (Diffusion decreases noise in polarization output and the extent of polarization), and Figure S3 (Image of microfluidics gradient labeled with tracer dye). Click here for file

Additional file 2 **Video S1**. Time-lapse video of *STE20-GFP bni1Δ *cells. *STE20-GFP bni1Δ *cells were treated with 20 μM α-factor and imaged over a 10 min period at 1 min intervals. The polarization of Ste20-GFP was not maintained in a single location as observed in wild-type cells, but instead shifted position. There were also multiple peaks of polarization. Click here for file
