# Supplementary material for: Noise filtering tradeoffs in spatial gradient sensing and cell polarization response
Source: BMC Syst Biol. 2011 Dec 13;5:196. doi: 10.1186/1752-0509-5-196 (PMC3268761; doi:10.1186/1752-0509-5-196)
Supplement: Additional file 1 — Supplemental Material. This file contains Table S1 (Yeast strains), a description of the mathematical models, Figure S1 (Parametric analysis of k0 and k1), Table S2 (Effect of gradient slope versus noise on polarization), Table S3 (Effects of noise on polarization quality), a section estimating external gradient noise, Table S4 (Effects of ligand diffusion noise (σL) and receptor-ligand binding noise (σRL) on projection directional accuracy), a comparison of chemotactic index to cos(θ) measure of directional accuracy, Table S5 (Effect of diffusion of the polarized species on polarization), Figure S2 (Diffusion decreases noise in polarization output and the extent of polarization), and Figure S3 (Image of microfluidics gradient labeled with tracer dye). [file 1752-0509-5-196-S1.PDF]

Additional file 1: Supplemental Material

Noise Filtering Tradeoffs in Spatial Gradient Sensing and Cell Polarization Response

Ching-Shan Chou[1], Lee Bardwell[3], Qing Nie[2,4], and Tau-Mu Yi[3,4,5]

chou@math.ohio-state.edu, bardwell@uci.edu, qnie@math.uci.edu, tmy@uci.edu

[1]Department of Mathematics,
The Ohio State University,
Columbus, OH 43210, USA

[2]Center for Mathematical and Computational Biology
Center for Complex Biological Systems
Department of Mathematics
University of California, Irvine
Irvine, CA 92697, USA

[3]Center for Complex Biological Systems
Department of Developmental and Cell Biology
University of California, Irvine
Irvine, CA 92697, USA

[4]Co-senior authors

[5]Corresponding author:
Tau-Mu Yi
Assistant Professor of Developmental and Cell Biology
2011 Biological Sciences III
University of California, Irvine
Irvine, CA 92697
tmy@uci.edu (email)
949-824-4888 (phone), 949-824-4709 (fax)

Table of Contents (Additional file 1, Supplemental Material)

1. Table S1. Yeast strains
2. Mathematical model descriptions
    2.1. One-stage generic model
    2.2. Two-stage generic model
    2.3. Yeast model of pheromone-induced cell polarity
3. Parametric analysis of k_0 and k_1
    3.1. Figure S1. Effects of varying k_0 and k_1 on noise tolerance and polarization
4. Relationship between polarization and the signal-to-noise ratio
    4.1. Table S2. Effect of gradient slope versus noise on polarization
5. Effects of noise on polarization quality
    5.1. Table S3. Effects of noise on polarization quality
6. Estimating external gradient noise for yeast cells
7. Effect of receptor-ligand binding noise on polarization
    7.1. Table S4. Effects of ligand diffusion noise (σ_L) and receptor-ligand binding noise (σ_{RL}) on projection directional accuracy
8. Comparison of chemotactic index (CI) to cos(θ) measure of directional accuracy
9. Diffusion of internal cellular components reduces output noise
    9.1. Table S5. Effect of diffusion of the polarized species on polarization
    9.2. Figure S2. Diffusion decreases noise in polarization output and the extent of polarization
10. Figure S3. Image of microfluidics gradient labeled with tracer dye

1. Table S1

Table S1. Yeast strains

Strain	Genotype	Source
RJD863	*MAT**a** can1-100 leu2-3,-112 his3-11,-15 trp1-1 ura3-1 ade2-1 bar1Δ::hisG*	Ray Deshaies
RJD415	*MAT**a** can1-100 leu2-3,-112 his3-11,-15 trp1-1 ura3-1 ade2-1 bar1::LEU2 pep4::TRP1*	Ray Deshaies
TMY230	RJD415 *STE20-GFP::HIS5*	This study
TMY231	RJD415 *bem1-299Δ::KAN^R STE20-GFP::HIS5*	This study
TMY232	RJD415 *bni1Δ::KAN^R STE20-GFP::HIS5*	This study

2. Mathematical model descriptions

2.1. One-stage generic model

$$\frac{\partial a}{\partial t} = D\nabla_s^2 a + \frac{k_0}{1+(\beta u)^{-q}} + \frac{k_1}{1+(\gamma a p)^{-h}} - \left(k_2 + k_3 b\right)a \quad \text{(S1-1)}$$

$$\frac{db}{dt} = k_4\left(\tilde{a} - k_{ss}\right)b \quad \text{(S1-2)}$$

$$\tilde{a} = \frac{\int_s a\, ds}{\int_s ds}$$

$$p = \frac{1}{1+(\beta u)^{-q}}$$

Gradient input and cell dimensions

$u = L_{mid} + L_{slope}(z - z_0)$; $L_{mid} = 1$, $L_{slope} = 0.001$ to 1 (μm)$^{-1}$
Cell geometry: 1 μm radius sphere

Rate constants

$k_0 = 10$, $k_1 = 0$ (no positive feedback, NPF)
$k_0 = 1$, $k_1 = 10$ (positive feedback, PF)
$k_2 = k_3 = k_4 = 1$
$q = 100$ or 1000
$h = 0, 2, 8$
$\beta = 1$, $\gamma = 1$
$k_{ss} = 1$
$D = 0.001\ \mu\text{m}^2$/s

Initial conditions

$a_0 = 1$, $b_0 = 1$

The spatial (polarized) variable is *a*, and the variable *b* is the negative regulator governed by the integral control equation (S1-2). The k_0 term represents an ultrasensitive

(cooperative) response to the ligand input u, and the k_1 term represents a positive feedback loop that contains a cooperative-dependence on a in the form of a Hill term [1]. The $\tilde{a}$ term in Eq. (S1-2) represents the average value of a over the cell surface, i.e. the integral of a over the cell surface divided by the total surface area.

2.2. Two-stage generic model

$$\frac{\partial a_1}{\partial t} = D\nabla_s^2 a_1 + \frac{k_{10}}{1+(\beta_1 u)^{-q_1}} + \frac{k_{11}}{1+(\gamma_1 a_1 p_1)^{-h_1}} - \left(k_2 + k_3 b_1\right) a_1 \quad \text{(S2-1)}$$

$$\frac{db_1}{dt} = k_4 \left(\tilde{a}_1 - k_{1ss}\right) b_1 \quad \text{(S2-2)}$$

$$\frac{\partial a_2}{\partial t} = D\nabla_s^2 a_2 + \frac{k_{20}}{1+(\beta_2 a_1)^{-q_2}} + \frac{k_{21}}{1+(\gamma_2 a_2 p_2)^{-h_2}} - \left(k_2 + k_3 b_2\right) a_2 \quad \text{(S2-3)}$$

$$\frac{db_2}{dt} = k_4 \left(\tilde{a}_2 - k_{2ss}\right) b_2 \quad \text{(S2-4)}$$

$$\tilde{a}_1 = \frac{\int_s a_1 \, ds}{\int_s ds}, \quad \tilde{a}_2 = \frac{\int_s a_2 \, ds}{\int_s ds}$$

$$p_1 = \frac{1}{1+(\beta_1 u)^{-q_1}}, \quad p_2 = \frac{1}{1+(\beta_2 a_1)^{-q_2}}$$

Rate constants

$k_{10} = 10, k_{11} = 0; k_{20} = 10, k_{21} = 0$ (NPF+NPF)
$q_1 = q_2 = 100; h_1 = 0, h_2 = 0$

$k_{10} = 10, k_{11} = 0; k_{20} = 1, k_{21} = 10$ (NPF+PF)
$q_1 = 2, q_2 = 100; h_1 = 0, h_2 = 8$

$\beta_1 = 1, \beta_2 = 1; \gamma_1 = \gamma_2 = 1$
$k_2 = k_3 = k_4 = 1$

$k_{1ss} = k_{2ss} = 1$
$D = 0.001\ \mu\text{m}^2/\text{s}$

In the two-stage model, the output of the first stage (a_1) is the input to the second stage. The output of the second stage (a_2) is the system output. For the fast and slow versions of the model, the parameters were scaled by a factor of 10. The $\tilde{a}_1$ (S2-2) and $\tilde{a}_1$ (S2-4) terms

respectively represent the integral of a_1 and a_2 over the cell surface divided by the total surface area.

2.3. Yeast model of pheromone-induced cell polarity

We used a model of yeast cell polarization induced by mating pheromone that has been previously described [1]. This model was based on the spatial dynamics of the heterotrimeric and Cdc42 G-protein cycles. Receptor (R) binds ligand (L) and becomes activated (RL). Activated receptor converts heterotrimeric G-protein (G) into activated α-subunit (Ga) and free Gβγ (Gbg). All of these species are on the membrane. The connection between the two cycles is through free Gβγ which recruits cytoplasmic Cdc24 to the membrane. Membrane-bound Cdc24 (C24m) activates Cdc42. Activated Cdc42 (C42a) recruits the scaffold protein Bem1 (B1) to the membrane. Finally, a positive feedback loop is created because membrane-bound Bem1 can bind and recruit more Cdc24 to the membrane.

In the *bem1-299Δ* mutant, $h = 0$ and $k'_{24cm1} = 0.1 \times k_{24cm1}$. In the *bni1Δ* mutant $p_s = 0$.

$$\frac{\partial[\mathrm{R}]}{\partial t} = D\nabla_s^2[\mathrm{R}] - k_{RL}[\mathrm{L}][\mathrm{R}] + k_{RLm}[\mathrm{RL}] - k_{Rd0}[\mathrm{R}] + p_s k_{Rs} \quad \text{(S3-1)}$$

$$\frac{\partial[\mathrm{RL}]}{\partial t} = D\nabla_s^2[\mathrm{RL}] + k_{RL}[\mathrm{L}][\mathrm{R}] - k_{RLm}[\mathrm{RL}] - k_{Rd1}[\mathrm{RL}] \quad \text{(S3-2)}$$

$$\frac{\partial[\mathrm{G}]}{\partial t} = D\nabla_s^2[\mathrm{G}] - k_{Ga}[\mathrm{RL}][\mathrm{G}] + k_{G1}[\mathrm{Gd}][\mathrm{Gbg}] \quad \text{(S3-3)}$$

$$\frac{\partial[\mathrm{Ga}]}{\partial t} = D\nabla_s^2[\mathrm{Ga}] + k_{Ga}[\mathrm{RL}][\mathrm{G}] - k_{Gd}[\mathrm{Ga}] \quad \text{(S3-4)}$$

$$\frac{\partial[\mathrm{Gbg}]}{\partial t} = D\nabla_s^2[\mathrm{Gbg}] + k_{Ga}[\mathrm{RL}][\mathrm{G}] - k_{G1}[\mathrm{Gd}][\mathrm{Gbg}] \quad \text{(S3-5)}$$

$$\frac{\partial[\mathrm{Gd}]}{\partial t} = D\nabla_s^2[\mathrm{Gd}] + k_{Gd}[\mathrm{Ga}] - k_{G1}[\mathrm{Gd}][\mathrm{Gbg}] \quad \text{(S3-6)}$$

$$\frac{\partial[\mathrm{C24m}]}{dt} = D\nabla_s^2[\mathrm{C24m}] + k_{24cm0}(\mathrm{Gbg}_\mathrm{n}^*)[\mathrm{C24c}] + k_{24cm1}(\mathrm{B1}^*)[\mathrm{C24c}] - k_{24mc}[\mathrm{C24m}] - k_{24d}[\mathrm{Cla4a}][\mathrm{C24m}] \quad \text{(S3-7)}$$

$$\frac{\partial[\mathrm{C42}]}{dt} = D\nabla_s^2[C42] - k_{42a}[\mathrm{C24m}][\mathrm{C42}] + k_{42d}[\mathrm{C42a}] \quad \text{(S3-8)}$$

$$\frac{\partial[\text{C42a}]}{dt} = D\nabla_s^2[\text{C42a}] + k_{42a}[\text{C24m}][\text{C42}] - k_{42d}[\text{C42a}] \quad \text{(S3-9)}$$

$$\frac{\partial[\text{B1m}]}{dt} = D\nabla_s^2[\text{B1m}] + k_{B1cm}[\text{C42a}][\text{B1c}] - k_{B1mc}[\text{B1m}] \quad \text{(S3-10)}$$

$$\frac{\text{d}[\text{Cla4a}]}{dt} = k_{Cla4a}(C42a_t^*) - k_{Cla4d}[\text{Cla4a}] \quad \text{(S3-11)}$$

$$\text{Gbg}_\text{n}^* = \frac{1}{1+\left(\delta(Gbg_n)\right)^{-q}}, \quad \delta = \frac{\text{SA}}{\int_s (Gbg_n)\, ds} \text{ and } (Gbg_n) = \frac{[\text{Gbg}]}{[\text{G}]_0}$$

$$\text{B1}^* = \frac{\text{B1}_t^*}{1+(\gamma\text{Gbg}_\text{n}^*[\text{B1m}])^{-h}}, \quad \text{B1}_t^* = \frac{\int_s [\text{B1m}]\, ds}{\text{SA}} \text{ and } \gamma = \frac{\text{SA}}{2\int_s [\text{B1m}]\, ds}$$

$$\text{C42a}_t^* = \frac{\int_s [\text{C42a}]\, ds}{\text{SA}}$$

$h = 8$ (wild-type), $h = 0$ (bem1-299Δ)

$q = 100$

Polarized synthesis of receptor

$$p_s = \frac{[\text{C42a}]}{C42a_t^*}, \text{ if } C42a_t^* > 0, \text{ else } p_s = 1$$

Conservation equations

$$\text{V}\cdot[\text{C24c}] = C24_t - \int_S [\text{C24m}]ds, \quad C24_t = 2000 \text{ mol}$$

$$\text{V}\cdot[\text{Blc}] = B1_t - \int_S [\text{Blm}]ds, \quad Bl_t = 3000 \text{ mol}$$

Gradient input and cell dimensions

$u = L_{mid} + L_{slope}(z - z_0)$; $L_{mid} = 10$ nM, $L_{slope} = 0.001$ to 1 nM $(\mu\text{m})^{-1}$

Cell geometry: 2 μm radius sphere

SA = 50.264 μm^2

V = 33.51 μm^3

Table of Rate Constants

Parameter value	Reaction	Rationale	Reference
$k_{RL} = 2\times10^{-3}$ (nM)$^{-1}$ s^{-1}	RL association	Measured	[2]
$k_{RLm} = 1\times10^{-2}$ s^{-1}	RL dissociation	Measured	[2]
$k_{Rd0} = 4\times10^{-4}$ s^{-1}<br>$k_{Rd1} = 4\times10^{-4}$ s^{-1}	R internalization	Fit to G-protein deactivation rate	[2]
$k_{Rs} = \frac{4 \text{ mol s}^{-1}}{\text{SA}}$	R synthesis	Ensure steady-state receptor number of 10,000/cell	[2]
$k_{Ga} = 1\times10^{-5}$ (mol)$^{-1}$ s^{-1} $\times$ SA	G-protein activation	Fit to G-protein activation and dose-response data	[2]
$k_{Gd} = 0.1$ s^{-1}	G-protein deactivation	Fit to G-protein activation and dose-response data	[2]
$k_{G1} = 1$ (mol)$^{-1}$ s^{-1} $\times$ SA	Heterotrimer association	Estimate (not rate-limiting)	[2]
$k_{42d} = 0.02$ s^{-1}	Cdc42 deactivation	From literature; 10 fold-slower than heterotrimeric G-protein. Consistent with polarization time-course and *bni1Δ* data	[3]
$k_{42a} = 1\times10^{-5}$ (mol)$^{-1}$ s^{-1} $\times$ SA	Cdc42 activation	To achieve proper steady-state levels of active Cdc42	[3]
$k_{24cm0} = 0.04$ s^{-1} $\times \frac{\text{V}}{\text{SA}}$	Gβγ recruitment of Cdc24	Equivalent of k_{20} in two-stage generic model; fit to produce proper polarization	
$k_{24cm1} = 3.3\times10^{-3}$ (mol)$^{-1}$ s^{-1} $\times$ V	Bem1 recruitment of Cdc24	Equivalent of k_{21} in two-stage generic model	
$k_{24mc} = 1$ s^{-1}	Cdc24, membrane to cytoplasm	Ensures fast Cdc24 dynamics relative to Cdc42	
$k_{B1mc} = 0.01$ s^{-1}	Bem1, membrane to cytoplasm	Same time-scale as Cdc42 deactivation rate	
$k_{B1cm} = 1\times10^{-5}$ (mol)$^{-1}$ s^{-1} $\times V$	Bem1, cytoplasm to membrane	To achieve proper Bem1 polarization	
$k_{Cla4a} = .006$ s^{-1}	Cla4 activation rate	Establish proper ratio of active Cla4	
$k_{Cla4d} = .01$ s^{-1}	Cla4 deactivation	Same time-scale as positive feedback loop	
$k_{24d} = \frac{\text{SA}}{3000}$ s^{-1}	Negative regulation of Cdc42 cycle	Negative feedback gain; ensures model stability/robustness	

The parameters can be divided into two groups: (1) Heterotrimeric G-protein parameters and (2) Cdc42 parameters. The first set of parameters was directly measured or was fit to G-protein activation/deactivation kinetics [2]. The time-scale was set by the G-protein deactivation rate. The Cdc42 dynamics were approximately 10-fold slower, and were dictated by the Cdc42 deactivation rate.

The Cdc24 and Bem1 dynamics formed the inner positive feedback loop and their parameters were tuned to ensure proper polarization of the model [1]. These constants represent the equivalent of k_{20} and k_{21} in the two-stage generic model.

The Cla4 dynamics represent in an approximate fashion the aggregate negative feedback regulation of Cdc42. This negative feedback loop was set to be on the same scale as the positive feedback loop, and the gain was adjusted to enhance model robustness.

The Hill constants $q = 100$ and $h = 8$ were the default values from the previous versions of the model [1]. Lower values i.e. $q = 10$ and $h = 2$ also produced polarization results similar to the default model.

Detailed individual parameter sensitivity analysis was not performed. Instead we scaled the two groups of parameters 10-fold up or 10-fold down to explore the effects of speeding up or slowing down the dynamics of each stage on the noise-resistance of the polarization (e.g. Fig. 3C).

Initial conditions

We may assume that [C42], [R], and [G] equally distributed along the surface with a total concentration $C42_t$, R_t and G_t respectively.

$$[\mathrm{R}]_0 = \frac{R_t}{\mathrm{SA}}\ , R_t = 10,000\ \text{mol (molecules)}$$

$$[\mathrm{G}]_0 = \frac{G_t}{\mathrm{SA}},\quad G_t = 10,000\ \text{mol}$$

$$[\mathrm{C42}]_0 = \frac{C42_t}{\mathrm{SA}},\ \ C42_t = 10,000\ \text{mol}$$

$$[\mathrm{RL}]_0 = 0,\ \ [\mathrm{Ga}]_0 = 0, [\mathrm{C24m}]_0 = 0, [\mathrm{C42a}]_0 = 0, [\mathrm{Blm}]_0 = 0$$

3. Parametric analysis of k_0 and k_1

We have performed parametric analysis in which we varied k_0 and k_1 (Fig. S1). We found that for a range of values there were two basic regimes: an NPF regime which produced reduced polarization ($a_f < 2$) that was more sensitive noise, and a PF regime which produced greater polarization ($a_f > 2$) that was less sensitive to noise. These regimes corresponded to the results for the NPF model in which input ultrasensitivity was the primary mode of amplification and for the PF model in which positive feedback was the primary mode of amplification, respectively, shown in Figures 1 and 2 (main text).

3.1. Figure S1

Figure S1. Effects of varying k_0 and k_1 on noise tolerance and polarization. In 1D simulations we tested the pairs (k_0, k_1) = (1, 10), (2, 8), (5, 5), (8, 2), (10, 0) for a fixed slope (L_{slp} = 0.01 μm^{-1}), three different noise values (σ= 0.01, 0.1, 1), and two values of q (q = 100, 1000). We calculated the mean value of a ($\overline{a}$) as a function of the axial length z. For higher k_1 values relative to k_0, the polarization was greater ($a_f > 2$) and less sensitive to noise. For higher k_0 values relative to k_1, the polarization was reduced ($a_f < 2$) and more sensitive to noise.

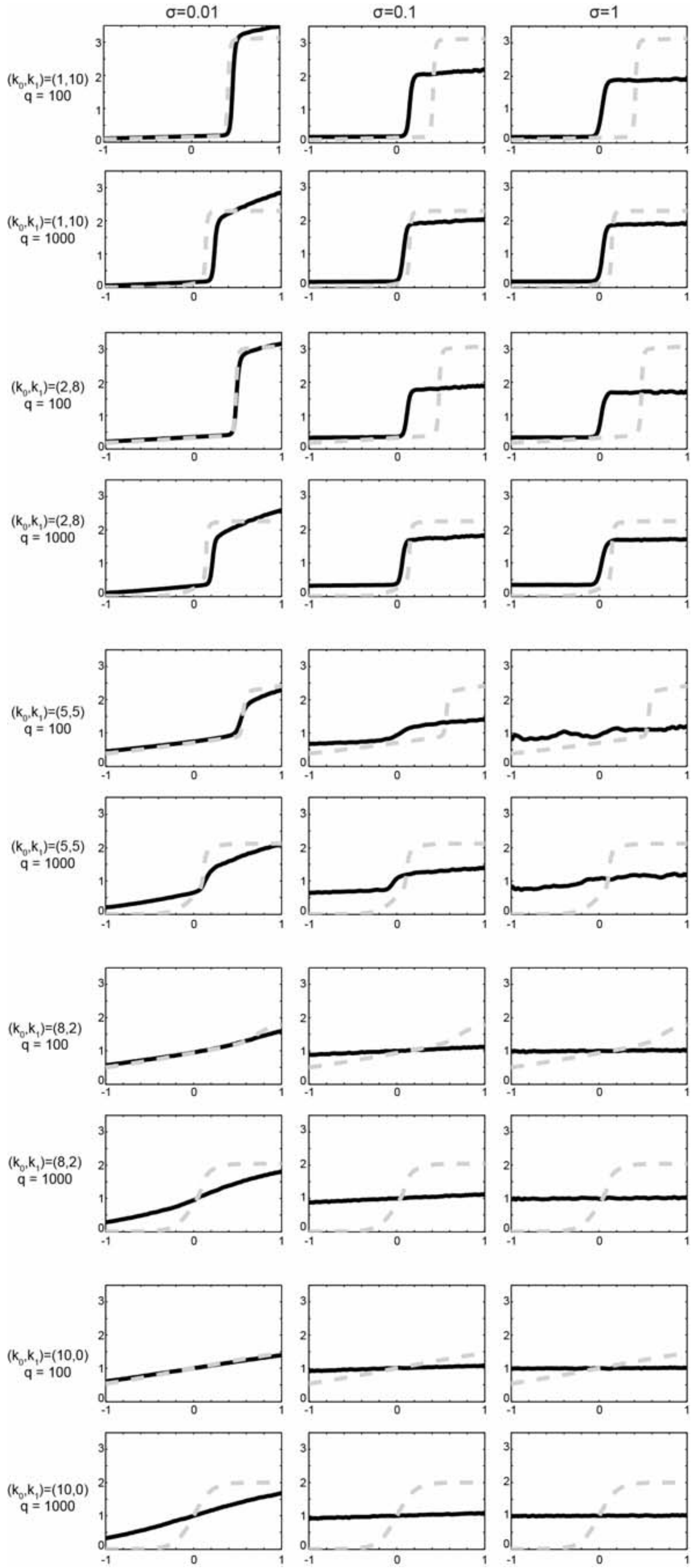

σ=0.01
σ=0.1
σ=1
(k0,k1)=(1,10) q = 100
(k0,k1)=(1,10) q = 1000
(k0,k1)=(2,8) q = 100
(k0,k1)=(2,8) q = 1000
(k0,k1)=(5,5) q = 100
(k0,k1)=(5,5) q = 1000
(k0,k1)=(8,2) q = 100
(k0,k1)=(8,2) q = 1000
(k0,k1)=(10,0) q = 100
(k0,k1)=(10,0) q = 1000

4. Relationship between polarization and the signal-to-noise ratio

There have been several proposed measures for signal-to-noise ratio (SNR) for spatial sensing and response [4-7]. All use the gradient slope as the signal, and some combination of factors as the noise term that represents ligand-diffusion, receptor-ligand binding, internal noise, etc. In our formulation, the standard deviation of the injected input noise is the noise term, which is a direct representation, and the signal-to-noise ratio is the gradient slope divided by this noise value. Thus, for example, there is no dependence on terms such as ligand concentration, which can have multiple effects on both the signal and the noise.

We then measured polarization in the generic no-positive-feedback model for different values of the SNR, varying both the gradient slope and the input noise. We assessed polarization by the mean value of the polarized component (a) at the front of the cell. The results are shown in Table S2.

For a given signal-to-noise ratio, we found that the degree of polarization was approximately constant, although this relationship broke down for larger gradient slopes. To show this relationship more clearly, we plotted polarization ($\overline{a}_f$) versus the signal-to-noise ratio for different gradient slopes except the highest in Table S2. Interestingly, there was an approximate linear relationship between polarization and the SNR as shown in Figure 1E (main text). The breakdown in this relationship occurred when the external gradient slope approached that of the internal slope of the polarized component. One interpretation is that the system no longer needed to amplify the external gradient to create the internal gradient, and as a result, the noise had less of an effect on the polarization.

4.1. Table S2

Table S2. Effect of gradient slope versus noise on polarization

σ	$\bar{a}_f$				
	$L_{slp} = 0.01$	$L_{slp} = 0.03$	$L_{slp} = 0.1$	$L_{slp} = 0.3$	$L_{slp} = 1$
$\sigma = 0.001$	2.00	2.00	2.00	2.00	2.00
$\sigma = 0.01$	1.66	2.00	2.00	2.00	2.00
$\sigma = 0.1$	1.09	1.23	1.68	2.04	2.02
$\sigma = 1$	1.01	1.04	1.13	1.38	2.06
$\sigma = 10$	1.02	1.02	1.06	1.25	1.70

The average value of the spatial variable a at the front of the cell (where the ligand concentration is maximum) is represented by $\bar{a}_f$. The no-positive-feedback (NPF) model with $q = 1000$ was used. L_{slp} is the gradient slope (μm^{-1}), and σ is the standard deviation of the noise.

5. Table S3

Table S3. Effects of noise on polarization quality

A. NPF model

	**cos(*θ*)**	a_f	σ_{out}
***q* = 10**	**1.0**	**1.39**	**0.04**
***q* = 100**	**1.0**	**1.67**	**0.079**
***q* = 1000**	**1.0**	**1.68**	**0.085**

$L_{slp} = 0.1\ \mu\text{m}^{-1},\ \sigma = 0.1$.

B. PF model

	**cos(*θ*)**	a_f	σ_{out}
***h* = 2**	**1.0**	**1.07**	**0.13**
***h* = 4**	**0.97**	**1.82**	**0.16**
***h* = 8**	**0.93**	**1.87**	**0.22**

$L_{slp} = 0.01\ \mu\text{m}^{-1},\ \sigma = 1$.

6. Estimating external gradient noise for yeast cells

From the theory [8], we obtained an estimate of the noise level (variance of ligand concentration c) in our simulations of the yeast system using the following formula: $\frac{\delta c}{\bar{c}} = \frac{1}{\sqrt{Dl\bar{c}\tau}}$. We estimated the parameters to be $D \sim 100$ μm^2/s (diffusion coefficient of ligand), $l \sim 0.1$ to 0.01 μm (length scale of receptor), $\bar{c} = 6$ (mean concentration of ligand), and $\tau = 0.01$ (integration time, set by simulation noise time step). For a single receptor, the appropriate length scale l ranges from the patch of membrane for each receptor (0.1 μm) to the molecular dimension of the receptor (0.01 μm). These values correspond to $\sigma \sim 1.3$ to 4.1. We were primarily concerned with ligand-diffusion noise as a lower-bound on the input noise encountered by the cell.

7. Effect of receptor-ligand binding noise on polarization

In this work, we primarily focused on ligand-diffusion noise which is external to the cell. However, there is also the noise created by the stochastic binding of ligand to receptor, receptor-ligand binding noise [9]. Both create fluctuations in the levels of the receptor-ligand complex (RL). We focused on the former because it is imposed by physical constraints, whereas the receptor-ligand binding noise can be affected by the properties of the receptor which can be considered "internal" to the cell. Bialek and Setayeshgar [8] have used the fluctuation dissipation theorem to decompose the uncertainty in the measurement of ligand into two terms:

$$\left(\frac{\delta c_{rms}}{c}\right)^2 = \frac{2}{mk_+\bar{c}(1-\bar{n})\tau} + \frac{1}{\pi D\bar{c}\tau}\left(\frac{\Lambda}{m} + \frac{\phi(m)}{2m}\right) \quad \text{(S4-1)}$$

The first term represents receptor-ligand binding noise and the second represents the ligand-diffusion noise. In the first term, *m* is the number of receptors in a cluster at each position, k_+ is the receptor-ligand association rate constant, $\bar{c}$ is the mean ligand concentration, $\bar{n}$ is the mean fractional occupancy of receptor, and τ is the integration time.

Some have argued that the receptor-ligand binding noise term is dominant; others have taken the point of view that it is worthwhile to investigate the hard limits imposed by the physics of ligand diffusion [5, 8, 10]. In this section, we wished to explore different combinations of these two noise sources.

A priori, there are three scenarios: (a) ligand diffusion noise is dominant, (b) they are approximately equal, or (c) receptor binding noise is dominant. Using the yeast model, we explored each of these scenarios. In our framework, the receptor-ligand binding noise

was introduced by adding white noise to the receptor-ligand association (k_{RL}) and dissociation (k_{RLm}) rate constants, which resulted in noise on RL levels. We found that the effect of the receptor-ligand binding noise was approximately equivalent to the ligand-diffusion noise in terms of its effect on directional accuracy (Table S4). For example, σ_L = 1 and σ_{RL} = 10 resulted in a directional accuracy of $\cos(\theta) = 0.29$, whereas σ_L = 10 resulted in $\cos(\theta) = 0.20$. In addition, σ_L = 1 and σ_{RL} = 1 resulted in a directional accuracy of $\cos(\theta) = 0.43$, whereas σ_L = 3 resulted in $\cos(\theta) = 0.29$. Thus, in the context of our simulation framework, we were able to explore the separate and combined effect of the two noise terms.

What is the approximate weighting of each term? Using the above formula (S4-1), we estimated that σ_{RL} is approximately 10. Thus, the total noise is in the range explored in Table S4. Interestingly, there is a dependence of σ_{RL} on the receptor cluster size, which is not known. Assuming that a lipid raft represents a cluster, then the cluster size $m \sim 100$ [11]; a larger cluster size would make the value of σ_{RL} smaller.

Finally, we note that in our generic models, the input noise in the simulations can be considered a combination of ligand-diffusion and receptor-binding noise because receptor is not explicitly represented. For the yeast model, we demonstrated that the combination is expected to increase the total noise on receptor-ligand (RL) levels, but still within the magnitude of noise values studied here.

In summary, it is likely that receptor-ligand binding noise has a significant effect and indeed is likely to be larger than the ligand noise. In the future, we would like to combine the two noise sources in a more realistic manner [12, 13] to study how they limit the accuracy and extent of yeast cell polarization.

7.1. Table S4

Table S4. Effects of ligand diffusion noise (σ_L) and receptor-ligand binding noise (σ_{RL}) on projection directional accuracy.

	cos(*θ*)			
	σ_{RL} = 0	σ_{RL} = 0.1	σ_{RL} = 1	σ_{RL} = 10
σ_L = 0	**1.00**			
σ_L = 1	**0.62**	**0.62**	**0.43**	**0.29**
σ_L = 10	**0.20**			

The filled gray boxes represent values not determined. Each entry represents the average $\cos(\theta)$ from over 30 Monte Carlo simulations of the yeast model ($L_{slp} = 0.01$ nM μm^{-1}) with the given values for σ_{RL} and σ_L.

8. Comparison of chemotactic index (CI) to cos(θ) measure of directional accuracy

The $\langle \cos(\theta) \rangle$ definition of the yeast projection direction (θ = angle formed between projection direction and gradient direction) is a commonly used measure of directional accuracy in the yeast community [14]. However, as the referee points out, this measure is not identical to the chemotactic index (*CI*). For motile cells, the chemotactic index is the distance traveled up the gradient divided by the total distance traveled. For a non-motile cell like yeast the equivalent chemotropic index (CI_{yeast}) is the total length of the projection divided by the length of the projection up the gradient. We compared CI_{yeast} with $\langle \cos(\theta) \rangle$ for a subset of cells in our experiments and found them to be similar: $CI_{yeast} - \langle \cos(\theta) \rangle = 0.05 \pm 0.03$.

9. Diffusion of internal cellular components reduces output noise

We explored whether enhanced diffusion of the internal components could attenuate noise and improve polarization. Using the generic no-positive-feedback (NPF) model and the generic positive feedback (PF) model, we increased the surface diffusion over a range of values and examined the polarization output. There was a striking effect of diffusion reducing the output noise, especially in the PF model. As a tradeoff, increased diffusion decreased the extent of polarization.

In the NPF model, we tested different diffusion constants D = 0, 0.001, 0.01, 0.1 $\mu m^2\ s^{-1}$. We also explored a range of noise values letting σ = 0, 0.01, 0.1, 1. We calculated the mean and standard deviation of a_f for each combination of D and σ (Table S5). The output noise, shown in the standard deviation (Std) column, decreased demonstrating the spatial smoothing effect of the higher level of diffusion (Fig S2A). Interestingly, there was little change in the extent of polarization. In the no noise case (σ = 0), increased diffusion resulted in only a slight decrease in polarization which was not apparent in a_f but in the overall shape of the polarization. On the other hand, diffusion did not preserve the extent of polarization in the presence of higher noise.

In the PF model, diffusion produced a striking effect. We explored diffusion values of D = 0, 0.001, 0.01, and 0.1 $\mu m^2\ s^{-1}$ with σ = 0 or 1. We ran 30 simulations for each combination of D and σ = 1, and calculated the output mean, standard deviation, as well as the directionality of the polarization ($\cos(\theta)$). Sample snapshots of the simulations are shown in Fig. S2C. In the absence of diffusion, the polarization was incoherent because of the noise with many sharp peaks; the presence of increasing diffusion resulted in smoother

polarization. Thus, the diffusion had a dramatic effect on reducing the output noise in the PF simulations.

As a tradeoff, diffusion caused a decrease in the extent of polarization in the PF model. In the $\sigma = 0$ case, there was a significant difference between the presence and absence of diffusion (Fig. S2B). In the noise case ($\sigma = 1$), there was also a loss of polarization at higher diffusion coefficients. For example when $D = 0.1$, the typical polarization was reduced, and more importantly, a subset of the simulations did not produce any polarization (green line), whereas at lower diffusion values, the simulations always produced some polarization (Fig. S2C).

Finally, increasing diffusion did not significantly affect directional accuracy $D > 0$: $\cos(\theta) = 0.67$ ($D = 0$), 0.93 ($D = 0.001$), 0.83 ($D = 0.01$), 0.85 ($D = 0.1$) Note that in the no diffusion case it was difficult to determine the polarization direction from the center-of-mass calculation.

9.1. Table S5

Table S5. Effect of diffusion of the polarized species on polarization

	**Output Polarization (a_f)**							
	***D* = 0**		***D* = 0.001**		***D* = 0.01**		***D* = 0.1**	
**Input noise**	**Mean**	**Std**	**Mean**	**Std**	**Mean**	**Std**	**Mean**	**Std**
$\sigma = 0$	**2.00**	**0.00**	**2.00**	**0.00**	**2.00**	**0.00**	**2.00**	**0.00**
$\sigma = 0.01$	**1.68**	**0.10**	**1.67**	**0.08**	**1.67**	**0.05**	**1.65**	**0.03**
$\sigma = 0.1$	**1.07**	**0.16**	**1.08**	**0.11**	**1.08**	**0.07**	**1.08**	**0.04**
$\sigma = 1$	**1.00**	**0.18**	**1.01**	**0.12**	**1.01**	**0.08**	**1.02**	**0.05**

The value of the spatial variable *a* at the front of the cell (where the ligand concentration is maximum) is represented by a_f. Both the mean and standard deviation (Std) of a_f are shown. The no-positive-feedback (NPF) model with q = 1000 was used. *D* is the surface diffusion coefficient for *a* ($\mu m^2\ s^{-1}$), and σ is the square root of the noise variance.

9.2. Figure S2

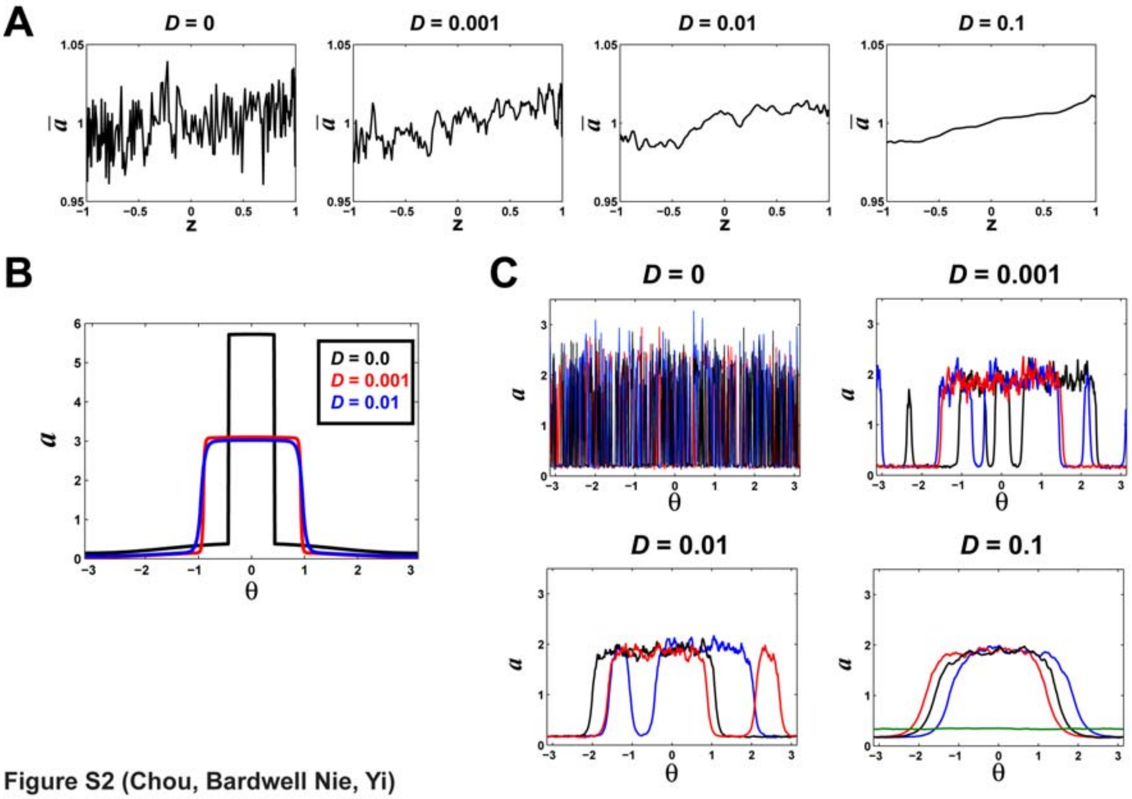

Figure S2 (Chou, Bardwell Nie, Yi)

Figure S2. Diffusion decreases noise in polarization output and the extent of polarization. In all simulations L_{mid} = 1, and L_{slp} = 0.01 μm^{-1}.

(A) Increasing diffusion reduced noise in the output of the NPF model. For a range of diffusion values (D = 0, 0.001, 0.01, 0.1) and with input noise σ = 1, we plotted the average value of a across the axial length z. The output was spatially smoother for larger diffusion coefficients.

(B) Diffusion decreased polarization in the absence of noise in the PF model. For different diffusion values, we plotted the polarization output in 2D simulations of the PF model. The polarization value a was plotted versus the radial coordinate θ (θ = 0 is the gradient direction). When D = 0.1, the simulation was unstable and underwent oscillations.

(C) Increasing diffusion reduced noise in the output and the extent of polarization in the PF model. From 30 2D simulations of the PF model at each diffusion coefficient value, 3 typical simulations (black, red, blue) were selected and displayed. For the $D = 0.1$ case, a fourth unpolarized simulation is shown, which represented approximately ¼ of all simulations.

10. Figure S3

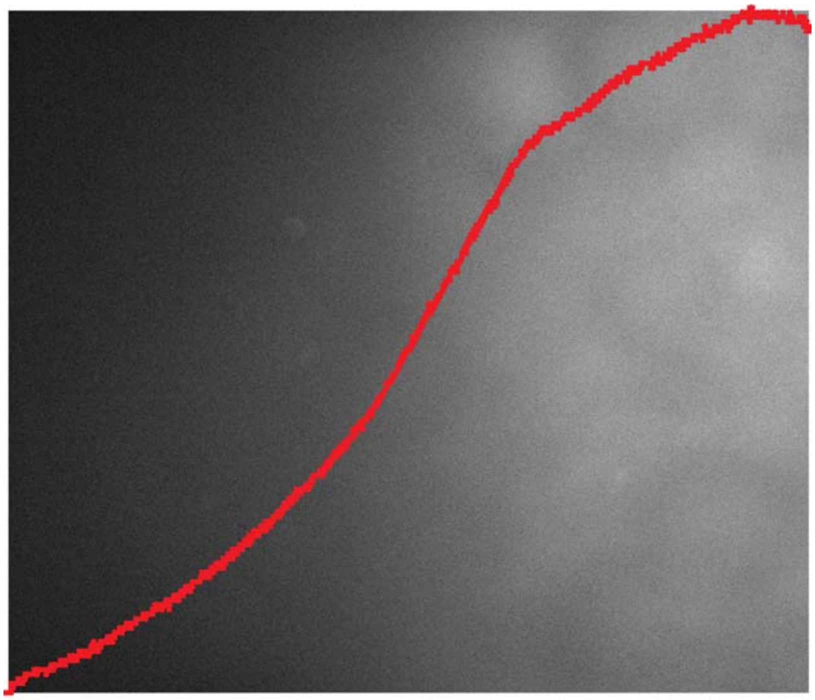

Figure S3 (Chou, Bardwell Nie, Yi)

Figure S3. Image of microfluidics gradient labeled with tracer dye. The image was taken at the position in the chamber in which the experiment was performed. The tracer Dextran-3000-TRITC diffused from right to left across the microfluidics device, and this diffusion was previously shown to be similar to the profile of α-factor labeled with Hylite-488 [15]. The fluorescence intensity was integrated across the image to create a concentration profile (red line) that was used to calculate the slope of the gradient.

REFERNCES (Supplemental Material)

1. Chou C-S, Nie Q, Yi T-M: **Modeling robustness tradeoffs in yeast cell polarization induced by spatial gradients.** *PLoS ONE* 2008, **3:**e3103.
2. Yi TM, Kitano H, Simon MI: **A quantitative characterization of the yeast heterotrimeric G protein cycle.** *Proc Natl Acad Sci U S A* 2003, **100:**10764-10769.
3. Leonard DA, Evans T, Hart M, Cerione RA, Manor D: **Investigation of the GTP-binding/GTPase cycle of Cdc42Hs using fluorescence spectroscopy.** *Biochemistry* 1994, **33:**12323-12328.
4. van Haastert PJM, Postma M: **Biased random walk by stochastic fluctuations of chemoattractant-receptor interactions at the lower limit of detection.** *Biophys J* 2007, **93:**1787-1796.
5. Endres RG, Wingreen NS: **Accuracy of direct gradient sensing by single cells.** *Proc Natl Acad Sci USA* 2008, **105:**15749-15754.
6. Mortimer D, Feldner J, Vaughan T, Vetter I, Pujic Z, Rosoff WJ, Burrage K, Dayan P, Richards LJ, Goodhill GJ: **A Bayesian model predicts the response of axons to molecular gradients.** *Proc Natl Acad Sci USA* 2009, **106:**10296 - 10301.
7. Fuller D, Chen W, Adler M, Groisman A, Levine H, Rappel W-J, Loomis WF: **External and internal constraints on eukaryotic chemotaxis.** *Proc Natl Acad Sci USA* 2010, **107:**9656 - 9659.
8. Bialek W, Setayeshgar S: **Physical limits to biochemical signaling.** *Proc Natl Acad Sci USA* 2005, **102:**10040-10045.
9. Lauffenburger DA, Linderman JJ: *Receptors: Models for Binding, Trafficking, and Signaling.* New York: Oxford University Press; 1993.
10. Berg HC, Purcell EM: **Physics of chemoreception.** *Biophys J* 1977, **20:**193-219.
11. Malinska K, Malinsky J, Opekarova M, Tanner W: **Distribution of Can1p into stable domains reflects lateral protein segregation within the plasma membrane of living *S. cerevisiae* cells.** *J Cell Sci* 2004, **117:**6031-6041.
12. Rappel W-J, Levine H: **Receptor noise limitations on cheotactic sensing.** *Proc Natl Acad Sci USA* 2008, **105:**19270 - 19275.
13. Endres RG, Wingreen NS: **Accuracy of direct gradient sensing by cell-surface receptors.** *Prog Biophys Mol Bio* 2009, **100:**33 - 39.
14. Segall JE: **Polarization of yeast cells in spatial gradients of alpha mating factor.** *Proc Natl Acad Sci U S A* 1993, **90:**8332-8336.
15. Moore TI, Chou CS, Nie Q, Jeon NL, Yi TM: **Robust spatial sensing of mating pheromone gradients by yeast cells.** *PLoS One* 2008, **3:**e3865.
